# Supplementary material for: Discontinuation of alpha-blocker therapy in men with lower urinary tract symptoms: a systematic review and meta-analysis
Source: BMJ Open. 2019 Nov 7;9(11):e030405. doi: 10.1136/bmjopen-2019-030405 (PMC6858108; doi:10.1136/bmjopen-2019-030405)
Supplement: Supplementary data [file bmjopen-2019-030405supp001.pdf]

Supplementary File 1. Search terms

Search terms

(Adrenergic alpha blockers [MeSH] OR Adrenergic alpha blockers OR alpha blockers OR doxazosin OR terazosin OR silodosin OR tamsulosin OR alfuzosin) AND (LUTS [MeSH] OR Prostatic Hyperplasia [MeSH] OR LUTS OR BPH OR lower urinary tract symptoms OR benign prostate hypertrophy OR benign prostate enlargement) AND (discontinu* OR interrup* OR cessa* OR stop* OR withdra* OR intermit*).

Supplementary File 2. Flow diagram of inclusion and exclusion of studies

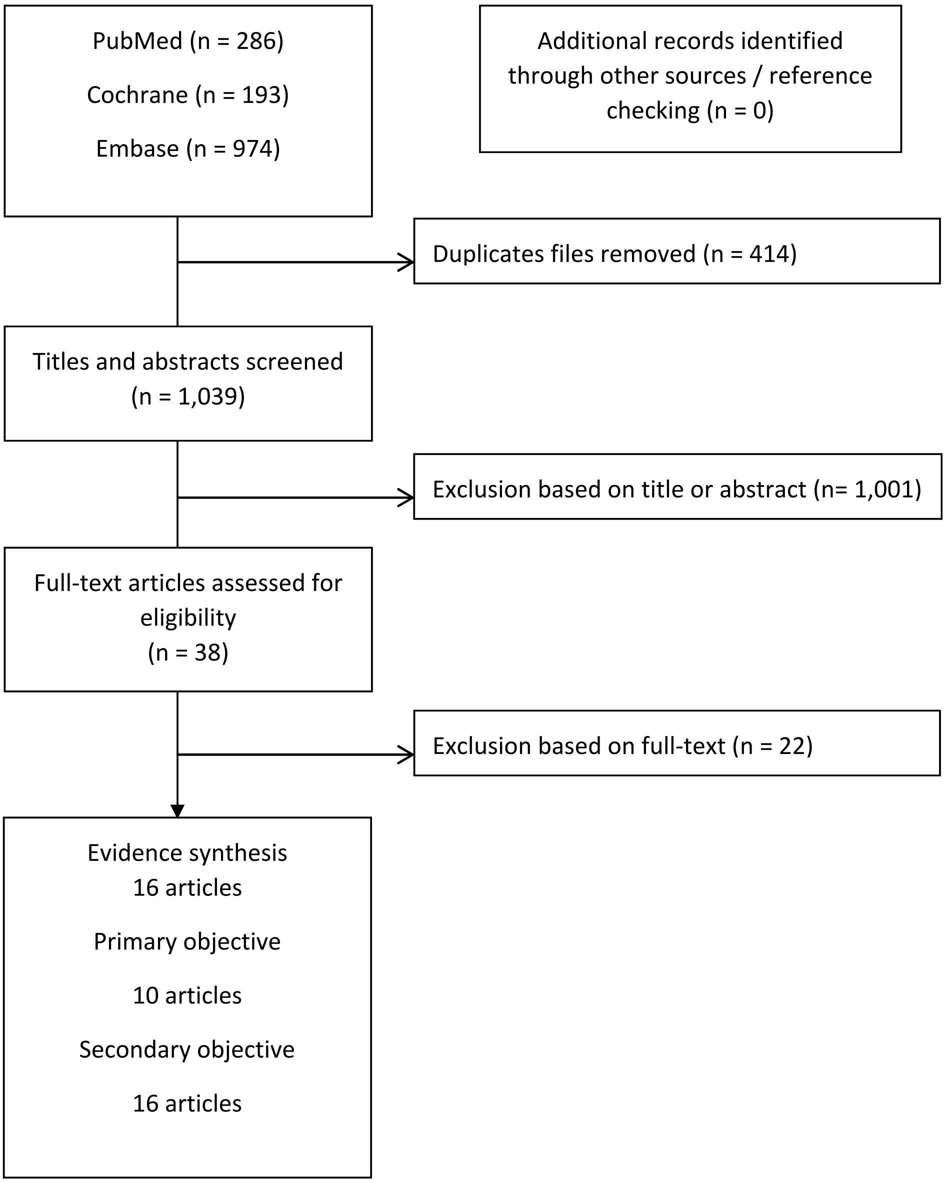

Supplementary File 3. Imputation of SD data from a previous meta-analysis

Tool	Ref.	Main author	Groups	N	SD	Pooled SD by study	Pooled SD overall
**IPSS**		**N = 12 studies**					
	49	Chapple	no data				
	73	Djavan	no data				
	147	Kirby	1	250	5.8	6.3	
			2	239	6.2		
			3	265	6.2		
			4	253	6.9		
	160	Lepor	no data				
	163	Lepor	no data				
	191	McConnell	no data				
	201	Mohanty	placebo	33	4	4.2	
			AB	36	4.4		
	208	Narayan	no data				
	254	Roehrborn	no data				
	255	Roehrborn	no data				
	262	Roehrborn	no data				
	305	van Kerrebroeck	N/A				
							**6.2**
**Q-max**		**N = 21 studies**					
	6	Abrams	N/A				
	33	Brawer	no data				
	50	chapple	no data				
	51	chapple	meta-analysis				
	53	christensen	N/A				
	79	elhilali	no data				
	101	gillenwater	no data				
	138	Kawabe	N/A				
	147	kirby	1	250	4.9		
			2	239	4.7	4.7	
			3	265	5.1		
			4	253	4.2		
	160	Lepor	no data				
	161	Lepor	no data				
	163	Lepor	no data				
	169	Lloyd	1	20	3.6		
			2	19	3.5	3.5	
			3	19	3.9		
			4	22	2.8		
	184	Martorana	N/A				
	201	Mohanty	placebo	33	2.6		
			AB	36	4.6	3.8	

	208	Narayan	no data			
	254	Roehrborn	no data			
	255	Roehrborn	no data			
	262	Roehrborn	no data			
	269	Schulman	N/A			
	305	van Kerrebroeck	N/A			
						**4.6**
**QoL**		**N = 5**				
	49	chapple	placebo	350	1	
			ocas 0.4	354	1.1	1.1
			mr 0.4	699	1.1	
			ocas 0.8	706	1.1	
	160	Lepor	no data			
	254	Roehrborn	no data			
	255	Roehrborn	placebo	763	1.1	
			alfuzosin	759	1.1	1.1
	305	van Kerrebroeck	N/A			
						**1.1**

In total, 12 studies assessed the IPSS, 21 assessed Q-max, and 5 assessed the IPSS QoL domain. Only studies that reported an SD at follow-up were included for the IPSS and Q-max data. No follow-up SDs were available for the QoL data, so baseline SDs were used. The SDs were imputed based on Table 6-35 from the NICE guideline on the management of LUTS [ref. 3].

Abbreviations: IPSS, International Prostate Symptom Score; N/A, article not accessible; Q-max, Peak urine flow rate; QoL, Quality of life; SD, standard deviation.

Supplementary File 4. Results of the risk of bias assessment

	Random sequence generation (selection bias)	Allocation concealment (selection bias)	Blinding of participants and personnel (performance bias)	Blinding of outcome assessment (detection bias)	Incomplete outcome data (attrition bias)	Selective reporting (reporting bias)	Other bias
Barkin et al	?	?	+	+	+	+	+
Debruyne et al	+	+	+	+	+	+	+
Fabricius	?	?	+	+	+	+	+
Gerber et al	−	−	−	−	−	+	+
Kaplan et al	−	−	−	−	+	+	+
Lee et al	+	+	−	−	−	+	+
Liaw & Kuo	−	?	−	−	?	+	?
Lin et al	+	?	−	−	+	+	+
Matsukawa	+	+	−	−	+	+	+
Yanardag et al	?	?	−	?	+	+	+
