# Supplementary material for: Discontinuation of alpha-blocker therapy in men with lower urinary tract symptoms: a systematic review and meta-analysis
Source: BMJ Open. 2019 Nov 7;9(11):e030405. doi: 10.1136/bmjopen-2019-030405 (PMC6858108; doi:10.1136/bmjopen-2019-030405)
Supplement: Author's manuscript [file bmjopen-2019-030405.draft_revisions.pdf]

BMJ Open

BMJ Open is committed to open peer review. As part of this commitment we make the peer review history of every article we publish publicly available.

When an article is published we post the peer reviewers' comments and the authors' responses online. We also post the versions of the paper that were used during peer review. These are the versions that the peer review comments apply to.

The versions of the paper that follow are the versions that were submitted during the peer review process. They are not the versions of record or the final published versions. They should not be cited or distributed as the published version of this manuscript.

BMJ Open is an open access journal and the full, final, typeset and author-corrected version of record of the manuscript is available on our site with no access controls, subscription charges or pay-per-view fees (http://bmjopen.bmj.com).

If you have any questions on BMJ Open's open peer review process please email info.bmjopen@bmj.com

BMJ Open

Discontinuation of alpha-blocker therapy in men with lower urinary tract symptoms: A systematic review and meta-analysis

Journal:	*BMJ Open*
Manuscript ID	bmjopen-2019-030405
Article Type:	Research
Date Submitted by the Author:	21-Mar-2019
Complete List of Authors:	van der Worp, Henk; University of Groningen, University medical center groningen, Department of General Practice and Elderly Care Medicine<br>Jellema, Petra; VU Amsterdam, 2. Department of Public and Occupational Health, Amsterdam Public Health research institute, Amsterdam UMC<br>Hordijk, Ilse; Isala Hospitals, urology<br>Lisman-van Leeuwen, Yvonne; University of Groningen, University medical center groningen, Department of General Practice and Elderly Care Medicine<br>Korteschiel, Lisa; Isala Hospitals, urology<br>Steffens, Martijn ; Isala Hospitals, urology<br>Blanker, Marco; University of Groningen, University medical center groningen, Department of General Practice and Elderly Care Medicine
Keywords:	alpha-blockers, discontinuation, lower urinary tract symptoms, meta-analysis

SCHOLARONE™
Manuscripts

Discontinuation of alpha-blocker therapy in men with lower urinary tract symptoms: A systematic review and meta-analysis

Henk van der Worp, PhD, post-doc researcher (0000-0001-5545-4155) [1], Petra Jellema, PhD, senior-researcher and epidemiologist [2], Ilse Hordijk, MD [3], Yvonne Lisman-van Leeuwen, PhD, epidemiologist [1], Lisa Korteschiel, MD [3], Martijn G Steffens, MD, PhD, urologist [3], Marco H Blanker, MD, PhD, GP and epidemiologist [1]

1. Department of General Practice and Elderly Medicine, University of Groningen, University Medical Centre Groningen, Groningen, the Netherlands
2. Department of Public and Occupational Health, Amsterdam Public Health research institute, Amsterdam UMC, Vrije Universiteit Amsterdam, the Netherlands
3. Department of Urology, Isala Clinics Zwolle, Zwolle, the Netherlands

Correspondence to:

Dr. Henk van der Worp

Department of General Practice and Elderly Medicine, University of Groningen, University Medical Centre Groningen, FA21, PO Box 196, 9700AD, Groningen, the Netherlands

h.van.der.worp@umcg.nl

+31 503615020

Word count: 3499

Keywords: alpha-blockers; discontinuation; lower urinary tract symptoms; meta-analysis

For peer review only - http://bmjopen.bmj.com/site/about/guidelines.xhtml

ABSTRACT

Objectives: We aimed to synthesize the available data for the effect of stopping alpha-blocker therapy among men with lower urinary tract symptoms. The focus was on symptom, uroflowmetry, and quality of life outcomes, but we also reviewed the adverse events and the number of patients who restarted therapy.

Eligibility criteria: We selected studies in which men were treated with an alpha-blocker for at least 3 months and in which the effects of alpha-blocker discontinuation were subsequently studied.

Information Sources: We searched MEDLINE/PubMed, EMBASE/Ovid, and The Cochrane Central Register of Controlled Trials from inception to May 2018

Risk of bias: Two reviewers independently assessed the risk of bias using the Cochrane Collaboration's tool for assessing risk of bias.

Included studies: We identified ten studies (1,081 participants) assessing the primary objective. Six studies (733 participants) assessed differences in adverse events between continuation and discontinuation and six studies (501 participants) reported the numbers of subjects that restarted treatment after discontinuation. No studies in primary care were identified.

Synthesis of the results: After discontinuing monotherapy, symptom scores increased and peak flow rates decreased at 3 and 6 months, but not at 12 months; however, neither parameter changed when alpha-blockers were stopped during combination therapy. Small differences in post-void residual volumes and quality of life scores were considered clinically irrelevant. We also found that adverse events did not increase with discontinuation and that 0%–49% of patients restarted after stopping alpha-blocker therapy.

Description of the effect: Discontinuing alpha-blocker monotherapy leads to a worsening

For peer review only - http://bmjopen.bmj.com/site/about/guidelines.xhtml

compared with continuing therapy. Discontinuing the alpha-blocker after combination therapy had no significant effects on outcomes in either the short or long term.

Interpretation: We conclude that discontinuation may be appropriate for the frail, elderly, or those with concomitant illness or polypharmacy. However, studies in primary care are lacking.

Registration: PROSPERO database (CRD42016032648)

https://www.crd.york.ac.uk/prospero/display_record.php?RecordID=32648

Strengths and limitations of the study:

- This is the first systematic review that synthesizes the literature concerning alpha-blocker discontinuation.
- The review was conducted in accordance with the PRISMA-guidelines.
- The number of studies that could be included was limited and the risk of bias was high for most outcomes preventing the drawing of firm conclusions.

Role of funding sources

This research received no specific grant from any funding agency in the public, commercial or not-for-profit sectors

Competing interest declaration

All authors have completed the ICMJE uniform disclosure form at www.icmje.org/coi_disclosure.pdf and declare: no support from any organisation for the submitted work; no financial relationships with any organisations that might have an interest in the submitted work in the previous three years; no other relationships or activities that could appear to have influenced the submitted work.

For peer review only - http://bmjopen.bmj.com/site/about/guidelines.xhtml

INTRODUCTION

Alpha-blockers are the first-choice treatment for men with moderate-to-severe lower urinary tract symptoms (LUTS) because of their proven, but small, superiority over placebo,[1-3] but their use can associated with dizziness, orthostatic hypotension, and increased fall risk.[3, 4] This may be especially problematic in the elderly, who often have polypharmacy and multi-morbidity. Given the natural course of LUTS, with 30% of patients showing improvement over time,[5] it may be appropriate to consider discontinuation of alpha-blocker therapy, especially in the elderly. There are no clear data on the effect of this approach but the guideline on male LUTS for Dutch general practitioners advises that alpha-blocker therapy be discontinued after 3–6 months, followed by symptom review.[2] By contrast, guidelines followed by urologists do not advocate routine discontinuation,[1, 6] though the European Association of Urology (EAU) do mention that alpha-blocker discontinuation may be considered after 6 months in the context of combination therapy.[1]

A number of researchers have studied the effects of discontinuing alpha-blockers, but to date, there has been no synthesis of this literature.

We performed a systematic review and meta-analysis to obtain data about the effect of discontinuing alpha-blockers on male LUTS. Our primary objective was to compare the effects of discontinuing therapy with those of continuing therapy. Secondary objectives were (1) to determine the proportion of men who restart alpha-blocker therapy and (2) to determine the possible adverse effects of both discontinuation and continuation.

METHODS

We completed this review according to the Preferred Reporting Items for Systematic Reviews and Meta-Analyses (PRISMA) and registered the protocol in the PROSPERO database (CRD42016032648) http://www.crd.york.ac.uk/PROSPERO/display_record.php?ID=CRD42016032648.

For peer review only - http://bmjopen.bmj.com/site/about/guidelines.xhtml

Selection criteria

We selected studies in which men were treated with an alpha-blocker for at least 3 months and in which the effects of alpha-blocker discontinuation were subsequently studied. For the primary objective, only randomized controlled trials (RCTs) and non-randomized controlled trials (NRCTs, including quasi-randomized trials) that compared alpha-blocker discontinuation to continuation were selected. For the secondary objectives, we also included uncontrolled studies. At all stages, we excluded studies written in languages other than Dutch, English, French, or German.

Outcome measures

The following outcomes were used for the primary objective: symptom scores, such as the International Prostate Symptom Score (IPSS); urinary flow rates; post-void residual urine volume (PVR); and quality of life (QoL). For the secondary objectives, we calculated the percentage of patients who restarted alpha-blocker therapy and the numbers of adverse events (AEs) in the continuation and discontinuation groups.

Search methods for identification of studies

We searched MEDLINE/PubMed, EMBASE/Ovid, and The Cochrane Central Register of Controlled Trials using search terms covering LUTS, alpha-blockers, and discontinuation (see Supplementary File 1 for detailed information). We ran the searches in January 2016 and updated them in July 2017 and May 2018. The reference lists of relevant articles were also screened to identify additional eligible studies. All duplicate files were removed before the titles and abstracts of the remaining records were independently screened by three reviewers [IH, LK, MB] and classified as "inclusion," "exclusion," or "uncertain." Next, the same reviewers independently applied the selection criteria to the full-text papers of all records classified into the inclusion or uncertain groups, and decided whether to include or exclude the research. Discrepancies in the selection procedure were resolved by consensus.

Data extraction process

Two authors [HW, IH] independently performed data extraction using standardized forms.

For peer review only - http://bmjopen.bmj.com/site/about/guidelines.xhtml

We extracted the following data: 1) the participant characteristics; 2) the interventions used; 3) the primary and secondary outcomes, as well as the timing of the outcome assessment; and 4) the study design. If possible, we extracted data by allocated intervention to allow an intention-to-treat analysis. Discrepancies were resolved by re-examination and discussion of the full-text papers or by consultation with a third author [MB].

Risk of bias

Two reviewers [HW, YL] independently assessed the risk of bias using the Cochrane Collaboration's tool for assessing risk of bias.[7] This tool includes six domains, as follows: selection bias, performance bias, detection bias, attrition bias, reporting bias, and other bias. Only RCTs and NRCTs were assessed, because these were used for the primary objective. Discrepancies in the risk of bias assessment were resolved by consensus or arbitration with a third party [MB]. Risk of bias was described for the different domains and summarized across studies and outcomes.[7]

Data analysis

Data were analyzed using Review Manager, Version 5.3.[8] We calculated the risk difference and 95% confidence intervals (CIs) for dichotomous variables and the mean differences (MD) with 95% CIs for continuous variables. For AEs, we also calculated the rate of AEs per 1,000 patient-days, based on the sample sizes and follow-up times.

Statistical heterogeneity was assessed by visual inspection of the forest plots and of the results of statistical testing for heterogeneity (I^2 statistic). We pooled data if we identified two or more studies with an I^2 of <40%,[9] using a random effects model. Data from both RCTs and NRCTs were pooled. Synthesizing and pooling were done separately for monotherapy and combination therapy. If the data for pooling were only presented in figures (e.g., standard deviations), it was extracted from those figures. If data was not present at all in the article, we contacted the authors if the article had been published in the past 10 years. If data could not be obtained in this way, we imputed data from a previous meta-analysis on the efficacy of alpha-blockers,[3] as described in the Cochrane Handbook.[9, 10]

For peer review only - http://bmjopen.bmj.com/site/about/guidelines.xhtml

Patient and public involvement

This study was performed without patient involvement. Patients were not invited to comment on the study design and were not consulted to interpret the results. Patients were not invited to contribute to the writing or editing of this document for readability or accuracy.

RESULTS

The searches yielded 1,039 publications (Supplementary File 2), of which 16 with a total of 1,823 participants were included (Table 1). All included studies were performed in secondary or tertiary care. Nine studies (772 participants) reported discontinuing alpha-blocker monotherapy: two double-blind RCTs,[11, 12] one open-label RCT,[13] two NRCTs,[14, 15] and four uncontrolled studies.[16-19] Six studies (980 participants) reported discontinuing alpha-blockers used in combination therapy: one double-blind RCT,[20] three open-label RCTs,[21-23] one NRCT,[24] and one uncontrolled study.[25] Finally, one uncontrolled study (N = 71) reported discontinuing both alpha-blocker monotherapy and combination therapy.[26]

Two of the included studies randomized patients into three groups: a discontinuation group, a continuation group, and a third group that continued with alternate-day use of alpha-blockers.[13, 14] We only used the data from the discontinuation and continuation groups.

In another three studies, the data required for pooling were missing.[11, 12, 20] Because these studies had been published over 15 years previously, no efforts were made to contact the authors. For one of the studies, means and standard deviations could be obtained from the figures.[12] For the other two studies,[11, 20] the standard deviations were missing and were imputed from the results of a previous meta-analysis (see Supplementary File 3).[3]

Risk of bias

Most of the included studies had risks of bias (Supplementary File 4). The most common were lack of blinding and randomization, with only three out of ten studies having a “low risk” for these items. There was no evidence of reporting bias in any of the included studies. The summary of

For peer review only - http://bmjopen.bmj.com/site/about/guidelines.xhtml

bias by study indicated that only one study had low risk of bias,[11] while two studies had unclear risks of bias,[12, 20] and the remaining studies had high risks of bias.[13-15, 21-24] As a result, risk of bias was high for all but one outcome.

Effects of alpha-blocker discontinuation

Five studies of monotherapy (n = 341) [11, 12, 14, 15] and five studies of combination therapy (n=740) [20-24] were used for the primary research objective. Only three provided data on the number of patients who did not comply with the intervention and who restarted alpha-blocker use after discontinuation. Two of these provided a per protocol analysis excluding those patients [23, 24] and the third provided an intention-to-treat analysis for categorized variables, with a per protocol analysis for the raw outcomes,[22] effectively precluding an intention-to-treat analysis. Because all included studies reported outcomes at 3, 6, or 12 months, we compared outcomes at these time points.

Symptom scores

All but one study [12] assessed symptoms with the IPSS questionnaire, or its predecessor the AUA symptom score (n = 1,054).

By 3 months after discontinuing alpha-blocker monotherapy, symptoms increased in the discontinuation group compared with the continuation group (MD = 4.17; 95% CI 2.91 to 5.43),[13, 14] whereas there was no difference between the continuation and discontinuation groups in the studies of combination therapy (MD = 0.97; 95% CI -0.32 to 2.27, Figure 1A).[20, 24]

After 6 months, two RCTs and one NRCT on monotherapy found a significant worsening of symptoms (differences varying from 2.0 to 5.8 points) in subjects that discontinued alpha-blockers.[11, 13, 14] No difference was found for studies on combination therapy after 6 months (MD = 0.56; 95% CI -1.57;2.69, Figure 1B).[23, 24]

After 12 months, the one study that looked at discontinuing monotherapy found a non-significant difference of 1.2 points between groups.[15] No differences were found in two open-label RCTs that looked at alpha-blocker discontinuation after combination therapy.[21, 22] Another NRCT

For peer review only - http://bmjopen.bmj.com/site/about/guidelines.xhtml

presented data for both groups, but did not make a direct statistical comparison between groups.[24] Data could not be pooled (I^2=61%).

Peak urine flow rate

Nine studies (804 patients) assessed peak urine flow rate (Q-max): five studies for monotherapy,[11-15] and four studies for combination therapy.[21-24]

After 3 months, Q-max reduced by 2.59 mL/s (95% CI 1.40 to 3.77, Figure 2A) in those who discontinued alpha-blocker monotherapy compared with those who continued therapy.[12-14] A single study on discontinuing combination therapy found that there was an increase of 1.4 mL/s in those who discontinued compared to those who continued therapy, but the researchers did not perform a statistical comparison.[24]

After 6 months, a reduction was again found in the Q-max after discontinuing monotherapy (MD = 1.79; 95% CI 0.73 to 2.86),[11, 13, 14] but no difference was found after discontinuing in the context of combination therapy (MD -0.23; 95% CI -1.51 to 1.05, Figure 2B).[23, 24]

After 12 months, no differences were found in an NRCT reporting on the effects of discontinuing monotherapy.[15] Three studies assessed Q-max 12 months after combination therapy. Among these, two open-label RCTs found no difference between groups:[21, 22] one study found a difference of 0.1 mL/s in favor of the continuation group and the other found that 7% fewer patients in the discontinuation group had a reduction in Q-max of >2 mL/s compared with the continuation group. Again, the NRCT on combination therapy showed an increase of 2.5 mL/s after alpha-blocker discontinuation, which was not seen in the group that continued alpha-blockers, but differences were not tested.[24] Data could not be pooled (I^2 = 68%).

Average urine flow rate (Q-avg)

Data from two RCTs on monotherapy (84 patients) could not be pooled (I^2 = 70%).[12, 13] After 3 months, one RCT reported a reduction of 2.2 mL/s in subjects who discontinued therapy compared with those who continued therapy,[12] whereas no statistical testing of the difference of 0.6 mL/s between groups was performed in the other RCT.[13]

For peer review only - http://bmjopen.bmj.com/site/about/guidelines.xhtml

After 6 months, this second study found a difference of 0.9 mL/s between groups in favor of continuing monotherapy.[13]

Post voided residual urine volume (PVR)

PVR volume was measured in five studies (n = 468), with three measuring it after discontinuing monotherapy [12, 13, 15] and two measuring it after discontinuing the alpha-blocker in combination therapy.[21, 22]

After 3 months, discontinuing monotherapy resulted in a PVR volume increase of 9.98 mL (95% CI 0.84 to 19.12, Figure 3).[12, 13]

After 6 months, an open-label trial on discontinuing monotherapy (57 participants) also found a statistically significant difference (14.1 mL) in favor of continuing therapy.[13]

At 12 months after discontinuing monotherapy, an NRCT did not find a significant difference between groups.[15] Two open-label RCTs on discontinuing combination therapy did report non-significant differences: one showed a 2 mL difference between groups,[21] and the other showed that 8% more patients in the discontinuation group reported a PVR increase of >50%.[22]

Quality of life

All five studies (one of monotherapy and four of combination therapy; 677 patients) that assessed QoL used the IPSS QoL sub-score.

After 3 months, one study of combination therapy found no difference between groups (0 points).[20] In another NRCT of combination therapy, a difference of 0.4 points was reported in favor of the group that discontinued therapy, but this was not statistically tested.[24]

After 6 months, one study on monotherapy found a statistically significant difference of 0.2 points in favor of those who continued alpha-blocker therapy.[11] This was the only outcome with a low risk of bias. A difference was found for the pooled studies of combination therapy (MD = 0.42; 95% CI 0.11–0.73, Figure 4).[23, 24]

After 12 months, no differences in QoL scores (only 0.1 points in favor of discontinuation) were found in an RCT of 117 participants receiving combination therapy.[21] Another NRCT of

For peer review only - http://bmjopen.bmj.com/site/about/guidelines.xhtml

patients receiving combination therapy found a difference of 0.4 in favor of continuation, but did not compare groups statistically.[24] Data could not be pooled (I^2 = 60%).

Restart of prior treatment and AEs

Patients restarting treatment

Six studies (501 patients) reported data on restarting alpha-blockers after discontinuation.[15, 16, 19, 22-24] In three of these (187 patients), 7%–49% of subjects restarted alpha-blockers after 6 months.[16, 19, 23] One of these,[19] together with another three studies (374 patients), [15, 22, 24] described that 0%–33% of subjects had restarted alpha-blockers at 12 months. The highest (49%) and lowest percentages (0%) restarting therapy were found in studies of monotherapy.[15, 16] However, four of the included studies explicitly advised subjects to restart alpha-blocker use if their PVR was >100 mL,[16, 19] or if symptoms worsened.[22, 24] These studies reported the highest restart rates.

Three other studies provided indirect information about restarting alpha-blocker use.[17, 25, 26] Two of these reported on successful discontinuation, defined as no increase in symptoms and no request for continuation of treatment. After 6 months, one indicated success among 69% of those receiving monotherapy.[17] Another study reported success rates of 13%–87% one month after discontinuing combination therapy, with percentages increasing as the duration of alpha-blocker use increased (ranging from 3 – 12 months).[25] Discontinuation was successful in 13%–20% of subjects who used alpha-blockers for 3 months and in 84%–87% of subjects who used them for 12 months. A third study stated that most patients whose symptoms worsened after discontinuation wished to restart their medication rather than undergo surgery.[26]

Adverse events

Nine studies provided no data on AEs during discontinuation, or if they did, provided data without a clear indication of the treatment group.[23] Another study only reported AEs during follow-up for those who discontinued alpha-blockers.[18] The six remaining studies reported 49 AEs

For peer review only - http://bmjopen.bmj.com/site/about/guidelines.xhtml

in 363 patients who discontinued alpha-blockers and 58 AEs in 370 patients who continued to use alpha-blockers.[11-14, 20, 21] The AE rates in patients who discontinued or continued alpha-blockers were 0.13 and 0.15 per 1,000 patient-days, respectively. The pooled data showed no risk difference for AEs when discontinuing or continuing either monotherapy (risk difference = -0.01; 95% CI -0.08 to 0.07) or combination therapy (risk difference = -0.03; 95% CI -0.07 to 0.01, Figure 5).

Respiratory tract infection and urinary retention were the two most common AEs after discontinuing alpha-blockers (11 studies in total), being reported in 1%–4% of patients [11, 20] and in 1%–3% of patients,[11, 21] respectively. The incidence of these AEs did not differ between groups.

DISCUSSION

The results of this systematic review indicate that discontinuing alpha-blocker monotherapy leads to a worsening of clinical symptoms and a decrease of urinary flow rates in the short-term (3–6 months) compared with continuing therapy. However, after one year, no differences were found in these or other outcomes. Discontinuing the alpha-blocker after combination therapy had no significant effects on outcomes in either the short or long term.

The worsening of symptoms over the short-term after stopping monotherapy was probably relevant to clinical practice. The reported differences in the IPSS between groups exceeded the minimal clinically important difference (MCID) of 2.7 points.[27] The difference in Q-max was also clinically relevant, exceeding the MCID of 2 mL/s after 3 months (between-group difference, 2.59 mL/s),[3] but not after 6 months (1.79 mL/s). The difference of 0.42 points in the QoL scores at 6 months after discontinuing combination therapy remained below the MCID of 0.5 points.[3] Although no MCID was available for PVR, we do not think that the reported mean difference of 10 mL after 3 months was clinically relevant.

The worsening of symptoms noted by 3–6 months after discontinuing monotherapy was larger than the reported improvement of symptoms after initiating therapy, which was reported to be 2.55 points (95% CI, 1.92–3.17) based on 12 RCTs with a total of 9,335 participants.[3] The

For peer review only - http://bmjopen.bmj.com/site/about/guidelines.xhtml

magnitude of change in the present review may have been influenced by the lack of blinding of both patients and assessors in many of the studies, which will have favored the continuation groups. Men in these studies who had no clear symptom improvements are likely to have dropped out before the discontinuation phase, so the participants subsequently included in the discontinuation trials will generally have had larger treatment effects and larger changes after discontinuation. The outcomes after 12 months relied on data from a single study on discontinuing doxazosin (not a controlled-release version),[15] which has a lower efficacy than other alpha-blockers. This might explain the lack of any meaningful long-term impact.

Among patients receiving combination therapy, outcomes were not significantly different between those discontinuing and continuing alpha-blockers. Although 5-alpha-reductase inhibitors have no significant impact on LUTS severity after treatment initiation,[28, 29] their continuation seems to be protective against symptom worsening after discontinuing alpha-blockers.

The results for restarting a discontinued alpha-blocker were heterogeneous, ranging widely from 0% to 49%. These conflicting findings can be explained by the differences in instructions given to patients in these studies. Indeed, participants in some studies received explicit instructions about when to restart therapy, whereas in other studies, no instructions were given. Also, subjects in cohort studies who volunteered to discontinue therapy may have had greater freedom to restart therapy than those participating in an RCT.

It was also shown that discontinuing alpha-blockers did not result in more AEs, including acute urinary retention.[30] Equally, continuation was not associated with more AEs, with neither dizziness nor orthostatic hypotension being more common.[4] This may be explained by subject drop-out due to AEs before entering the discontinuation phase. The number of patients reporting AEs in the included studies was, however, too small to draw meaningful conclusions regarding AEs.

Interpretation of our findings is hampered by some limitations. For example, the limited numbers of studies and large amount of statistical heterogeneity limited data pooling. We therefore decided to include both RCTs and NRCTs when pooling data, but the NRCT data may have introduced

For peer review only - http://bmjopen.bmj.com/site/about/guidelines.xhtml

selection bias. The limited number of RCTs also precluded sensitivity analyses. In addition, some studies gave unclear data about treatment compliance or only presented per protocol analyses, which may have led to bias (e.g., drop-out due to severe complaints) and loss of generalizability. Another issue is that all studies were performed in secondary or tertiary care settings. This is important if we consider that in some countries, most men with LUTS are treated in primary care. The high risk of bias, which was noted for all but one outcome, also hampers the interpretation of our findings. Finally, two of the trials of combination therapy compared discontinuing alpha-blockers and 5-alpha-reductase inhibitors, but the others compared discontinuing and continuing only the alpha-blocker.[22, 24]

No firm conclusions can be drawn from this review because of the low quality of the available evidence. Overall, the data suggest that there is a short-term clinical worsening of LUTS after discontinuing alpha-blocker monotherapy, as assessed by symptom scores and urinary flow rates, but that this does not increase the risk of a complicated symptom course.

Patients frequently discontinue alpha-blocker treatment in clinical practice. We have recently shown that men who continue to use alpha-blockers are typically unconcerned about stopping that therapy if advised to do so by a doctor.[31] The present review also provides evidence that the magnitude of symptom deterioration is limited, indicating that physicians can change their prescribing policy without risking harm. Indeed, the alternative approach may promote unnecessary polypharmacy, which is especially relevant in vulnerable groups. Active follow-up should then be used to monitor the need to restart alpha-blockers if symptoms worsen.

Our findings support the existing EAU guidance to consider discontinuing alpha-blockers in patients receiving combination therapy for 6 months.[1] Unfortunately, because the studies in this review were only performed in secondary care, we cannot give firm support for the recommendation of the Dutch GP guideline to review therapy after 3–6 months in primary care.[2] Symptom levels before treatment are generally lower in primary care, where conditions are typically less severe than in secondary care. Although the data from this review may be applicable to primary care, further

For peer review only - http://bmjopen.bmj.com/site/about/guidelines.xhtml

efficacy studies and discontinuation trials are needed to assess the outcomes specific to this setting.

Acknowledgments

We thank Dr Robert Sykes (www.doctored.org.uk) for providing editorial services.

Author statement

The corresponding author attests that all listed authors meet authorship criteria and that no others meeting the criteria have been omitted.

MHB had the idea for the article. Acquisition of the data was done by IH, LK, HW and MHB. Analysis and interpretation of the data was done by HW, MHB, PJ, YL and MGS. HW and MHB wrote the manuscript. All authors critically reviewed the manuscript.

Data sharing statement

Data collected for this study will be available from the corresponding author upon request.

For peer review only

References

1 Gratzke C, Bachmann A, Descazeaud A, et al. EAU Guidelines on the Assessment of Non-neurogenic Male Lower Urinary Tract Symptoms including Benign Prostatic Obstruction, *Eur Urol* 2015;67:1099-109.

2 Blanker M, Klomp M, Donk M, et al. Summary of the NHG practice guideline 'Lower urinary tract symptoms in men', *Ned Tijdschr Geneeskd* 2013;157:A6178.

3 National Clinical Guideline Centre (NICE).
The management of lower urinary tract symptoms in men: NICE guideline. 2010;CG97(01/09).

4 Bird ST, Delaney JA, Brophy JM, et al. Tamsulosin treatment for benign prostatic hyperplasia and risk of severe hypotension in men aged 40-85 years in the United States: risk window analyses using between and within patient methodology, *BMJ* 2013;347:f6320.

5 Kok ET, Bohnen AM, Groeneveld FP, et al. Changes in disease specific and generic quality of life related to changes in lower urinary tract symptoms: the Krimpen study, *J Urol* 2005;174:1055-8.

6 McVary KT, Roehrborn CG, Avins AL, et al. Update on AUA guideline on the management of benign prostatic hyperplasia, *J Urol* 2011;185:1793-803.

7 Higgins JP, Altman DG, Gotzsche PC, et al. The Cochrane Collaboration's tool for assessing risk of bias in randomised trials, *BMJ* 2011;343:d5928.

8 Review Manager (RevMan) [Computer program]. Version 5.3. Copenhagen: The Nordic Cochrane Centre, The Cochrane Collaboration, 2014.

9 Higgins JPT, Green S (editors). Cochrane Handbook for Systematic Reviews of Interventions Version 5.1.0 [updated March 2011]. The Cochrane Collaboration 2011. Available from www.handbook.cochrane.org.

10 Furukawa TA, Barbui C, Cipriani A, et al. Imputing missing standard deviations in meta-analyses can provide accurate results, *J Clin Epidemiol* 2006;59:7-10.

11 Debruyne FM, Witjes WP, Fitzpatrick J, et al. The international terazosin trial: a multicentre study of the long-term efficacy and safety of terazosin in the treatment of benign prostatic hyperplasia. The ITT Group, *Eur Urol* 1996;30:369-76.

12 Fabricius P G PG. Efficacy of once-a-day terazosin in benign prostatic hyperplasia: a randomized, double-blind placebo-controlled clinical trial. *Prostate.Suppl* 1990;3:85-93.

13 Yanardag H, Goktas S, Kibar Y, et al. Intermittent tamsulosin therapy in men with lower urinary tract symptoms, *J Urol* 2005;173:155-7.

14 Kaplan SA, Reis RB, Cologna A, et al. Intermittent alpha-blocker therapy in the treatment of men with lower urinary tract symptoms, *Urology* 1998;52:12-6.

For peer review only - http://bmjopen.bmj.com/site/about/guidelines.xhtml

15 Gerber GS, Contreras BA, Zagaja GP, et al. Doxazosin in men with lower urinary tract symptoms: urodynamic evaluation at 15 months, *Urology* 1997;50:229-33.

16 Chung JH, Lee JY, Kang DH, et al. Evaluation of patient outcome after discontinuation of alfuzosin treatment for benign prostatic hyperplasia: a multicentre, prospective study, *Int J Clin Pract* 2013;67:870-5.

17 Kobayashi M, Tokue A, Morita T. Discontinuation of tamsulosin treatment in men with lower urinary tract symptoms: a pilot study, *Urol Int* 2006;76:304-8.

18 Nickel JC, Barkin J, Koch C, et al. Finasteride monotherapy maintains stable lower urinary tract symptoms in men with benign prostatic hyperplasia following cessation of alpha blockers, *Can Urol Assoc J* 2008;2:16-21.

19 Yokoyama T, Watanabe T, Saika T, et al. Natural course of lower urinary tract symptoms following discontinuation of alpha-1-adrenergic blockers in patients with benign prostatic hyperplasia, *Int J Urol* 2007;14:598-601.

20 Barkin J, Guimaraes M, Jacobi G, et al. Alpha-blocker therapy can be withdrawn in the majority of men following initial combination therapy with the dual 5alpha-reductase inhibitor dutasteride, *Eur Urol* 2003;44:461-6.

21 Matsukawa Y, Takai S, Funahashi Y, et al. Effects of Withdrawing alpha1-Blocker from Combination Therapy with alpha1-Blocker and 5alpha-Reductase Inhibitor in Patients with Lower Urinary Tract Symptoms Suggestive of Benign Prostatic Hyperplasia: A Prospective and Comparative Trial Using Urodynamics, *J Urol* 2017;198:905-12.

22 Lin VC, Liao CH, Kuo HC. Progression of lower urinary tract symptoms after discontinuation of 1 medication from 2-year combined alpha-blocker and 5-alpha-reductase inhibitor therapy for benign prostatic hyperplasia in men--a randomized multicenter study, *Urology* 2014;83:416-21.

23 Lee JY, Kang DH, Park SY, et al. Effect of Discontinuation of Tamsulosin in Korean Men with Benign Prostatic Hyperplasia Taking Tamsulosin and Dutasteride: An Open-Label, Prospective, Randomized Pilot Study, *Low Urin Tract Symptoms* 2012;4:35-40.

24 Liaw Y-, Kuo H-. Discontinuation of an alpha-1 blocker or 5-alpha-reductase inhibitor after combination medical treatment in patients with benign prostatic hyperplasia, *Tzu Chi Med J* 2006;18:91-6.

25 Baldwin KC, Ginsberg PC, Roehrborn CG, et al. Discontinuation of alpha-blockade after initial treatment with finasteride and doxazosin in men with lower urinary tract symptoms and clinical evidence of benign prostatic hyperplasia, *Urology* 2001;58:203-9.

26 Kuo HC. Comparative study of therapeutic effect of dibenyline, finasteride, and combination drugs for symptomatic benign prostatic hyperplasia, *Urol Int* 1998;60:85-91.

27 Barry MJ, Williford WO, Chang Y, et al. Benign prostatic hyperplasia specific health status measures in clinical research: how much change in the American Urological Association symptom index and the benign prostatic hyperplasia impact index is perceptible to patients? *J Urol* 1995;154:1770-4.

For peer review only - http://bmjopen.bmj.com/site/about/guidelines.xhtml

28 Park T, Choi JY. Efficacy and safety of dutasteride for the treatment of symptomatic benign prostatic hyperplasia (BPH): a systematic review and meta-analysis, *World J Urol* 2014;32:1093-105.

29 Kirby RS, Roehrborn C, Boyle P, et al. Efficacy and tolerability of doxazosin and finasteride, alone or in combination, in treatment of symptomatic benign prostatic hyperplasia: the Prospective European Doxazosin and Combination Therapy (PREDICT) trial, *Urology* 2003;61:119-26.

30 Roehrborn CG. Reporting of acute urinary retention in BPH treatment trials: importance of patient follow-up after discontinuation and case definitions, *Urology* 2002;59:811-5.

31 Edelman M, Blanker M, Jellema P, et al. Patient attitudes towards deprescribing of alpha-blockers and willingness to participate in a discontinuation trial, *Neurourol Urodyn* 2017;36:S242-4.

For peer review only

Figure Captions

Figure 1. Forest plots of the IPSS when discontinuing or continuing alpha-blockers

A. Forest plot of the IPSS after 3 months for alpha-blocker discontinuation or continuation. B. Forest plot of the IPSS after 6 months for alpha-blocker discontinuation or continuation. Abbreviations: IPSS, International Prostate Symptom Score.

Figure 2. Forest plots of the Q-max when discontinuing or continuing alpha-blockers

A. Forest plot of the Q-max 3 months after alpha-blocker discontinuation or continuation. B. Forest plot of the Q-max 6 months after alpha-blocker discontinuation or continuation. Abbreviations: Q-max, peak urine flow rate.

Figure 3. Forest plot of the PVR 3 months after discontinuing or continuing alpha-blockers

Abbreviations: PVR, Post-void residual volume.

Figure 4. Forest plot of the QoL score 6 months after discontinuing or continuing alpha-blockers

Abbreviations: QoL, quality of life.

Figure 5. Forest plot of AEs after discontinuing or continuing alpha-blockers

Abbreviations: AEs, adverse events.

For peer review only - http://bmjopen.bmj.com/site/about/guidelines.xhtml

Table 1. Characteristics of the controlled and uncontrolled studies of monotherapy and combination therapy

Authors	Design	Type of AB	AB Use (Before Stopping)	No. Patients (Stop Phase)	Age (Mean)	IPSS at Baseline (Mean)	Measured Outcomes Primary	Measured Outcomes Secondary	Follow-Up (Months)
**Controlled trials**									
Monotherapy									
Fabricius et al. 1990 [12]	Double-blind RCT	TER	24 wk	27	68	-	Q-max, Q-avg, PVR,	AEs	3
Debruyne et al. 1996 [11]	Double-blind RCT	TER	26 wk	167	63.6	19.1	IPSS, Q-max, QoL	AEs	6
Gerber et al. 1997 [15]	NRCT	DOX	3 mo	37	65	C=20.9; DC=21.5	IPSS	Restart	12
Kaplan et al. 1998 [14]	NRCT	ALF	3 mo	53	60.5	15.6	IPSS, Q-max	AEs	3/6
Yanardag et al. 2005 [13]	Open-label RCT	TAM	3 mo	57	61.3	12.3	IPSS, Q-max, Q-avg, PVR	AEs	3/6
Combination therapy									
Barkin et al. 2003 [20]	Double-blind RCT	TAM	24 wk	277	C=67.6; DC=66.9	C=16.4; DC=16.5	IPSS, QoL	AEs	3
Liaw & Kuo 2006 [24]	NRCT	TAM	1 yr	47	C=70.7; DC=72.1	15.6	IPSS, Q-max, QoL	Restart	3/6/12
Lee et al. 2012 [23]	Open-label RCT	TAM	48 wk	69	68	15.3	IPSS, Q-max, QoL	Restart, AEs	6
Lin et al. 2014 [22]	Open-label RCT	DOX	2 yrs	230	75	C=13.1; DC=15.6	IPSS, Q-max, PVR	Restart	12
Matsukawa et al. 2017 [21]	Open-label RCT	SIL	12 mo	117	C=70.1; DC=69.1	C=17.4; DC=17.2	IPSS, Q-max, PVR, QoL	AEs	12
**Uncontrolled studies**									
Monotherapy									
Kobayashi et al. 2006 [17]	CS	TAM	28.5 ± 26.8 mo	33	70.4	16.3		Restart	6
Yokoyama et al. 2007 [19]	CS	NAF/ TAM /URA	2–200 mo	60	70 (median)	15.9		Restart	12
Nickel et al. 2008 [18]	CS	ALF/DOX/ TAM /TER	9 mo	220	66.1 (total sample)	19.9		Restart	9
Chung et al. 2013 [16]	CS	ALF	12 wk	58	68.6 (total sample)	16.7 (total sample)		Restart	6
Combination therapy									
Baldwin et al. 2001 [25]	CS	DOX	3–12 mo	240	66 (total sample)	Range: 20–33 (total study sample)		Restart	1
*Both**									
Kuo 1998 [26]	CS	DIB	6 mo	ABM=71; ABC=65	ABM=66.3; ABC=66.8	ABM=21.2; ABC=22.5		Restart	1

AB, alpha-blocker; ABC, alpha-blocker combination treatment; ABM, alpha-blocker monotherapy; AEs, adverse events; ALF = Alfuzosin; C, continuation group; CS, cohort

For peer review only - http://bmjopen.bmj.com/site/about/guidelines.xhtml

study; DC, discontinuation group; DIB = Dibenyline; DOX = Doxazosin; NAF = Naftopidil; NRCT, non-randomized controlled trial; ns, not stated; PVR, post voided residual urine volume; Q-avg, average urine flow rate; Q-max, peak urine flow rate; SIL = Silodosin; TAM = Tamsulosin; TER = Terazosin; URA = Urapidil.

* both monotherapy and combination therapy discontinuation

For peer review only

For peer review only - http://bmjopen.bmj.com/site/about/guidelines.xhtml

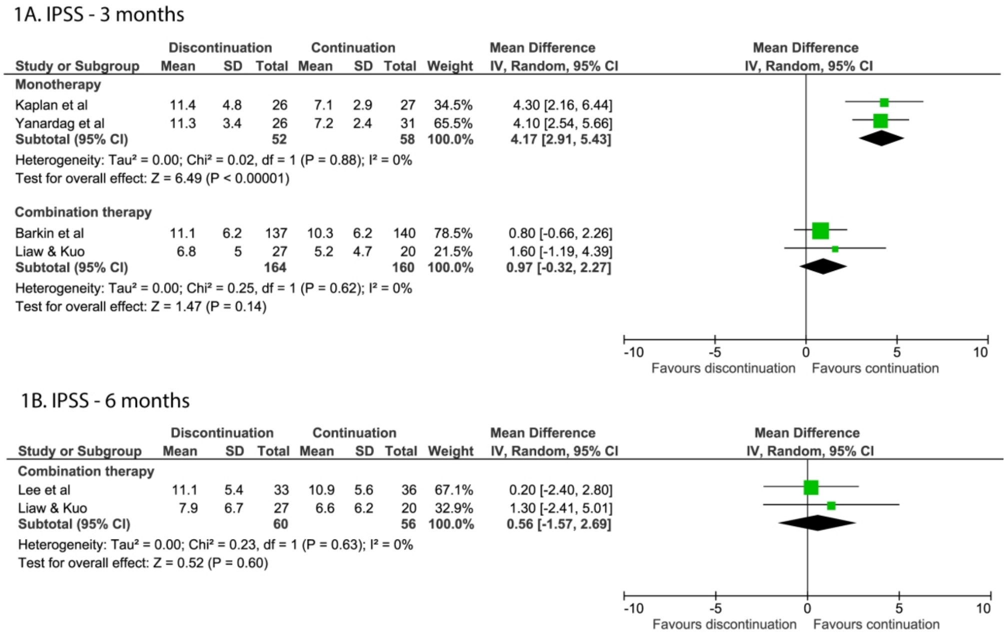

Figure 1. Forest plots of the IPSS when discontinuing or continuing alpha-blockers

2A. Q-max - 3 months

Study or Subgroup	Continuation Mean	Continuation SD	Continuation Total	Discontinuation Mean	Discontinuation SD	Discontinuation Total	Weight	Mean Difference IV, Random, 95% CI
**Monotherapy**								
Fabricius	13.9	3	13	10.4	3	14	27.4%	3.50 [1.24, 5.76]
Kaplan et al	12.7	4.8	27	9.7	2.5	26	33.5%	3.00 [0.95, 5.05]
Yanardag et al	11.3	4	31	9.7	3.3	26	39.1%	1.60 [-0.30, 3.50]
**Subtotal (95% CI)**			**71**			**66**	**100.0%**	**2.59 [1.40, 3.77]**

Heterogeneity: Tau² = 0.00; Chi² = 1.82, df = 2 (P = 0.40); I² = 0%
Test for overall effect: Z = 4.28 (P < 0.0001)

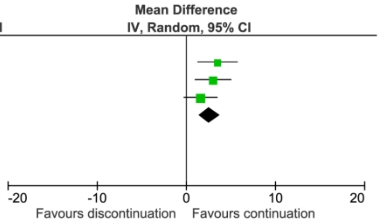

2B. Q-max - 6 months

Study or Subgroup	Continuation Mean	Continuation SD	Continuation Total	Discontinuation Mean	Discontinuation SD	Discontinuation Total	Weight	Mean Difference IV, Random, 95% CI
**Monotherapy**								
Debruyne et al	13.1	4.6	84	12.1	4.6	83	47.5%	1.00 [-0.40, 2.40]
Kaplan et al	11.7	5.2	27	9.3	2.1	26	23.0%	2.40 [0.28, 4.52]
Yanardag et al	11.1	4	31	8.5	3.1	26	29.5%	2.60 [0.76, 4.44]
**Subtotal (95% CI)**			**142**			**135**	**100.0%**	**1.79 [0.73, 2.86]**
Heterogeneity: Tau² = 0.12; Chi² = 2.29, df = 2 (P = 0.32); I² = 13%								
Test for overall effect: Z = 3.30 (P = 0.0010)								
**Combination therapy**								
Lee et al	10.4	3.3	36	10.7	2.9	33	76.9%	-0.30 [-1.76, 1.16]
Liaw & Kuo	11.1	4.9	20	11.1	4.2	27	23.1%	0.00 [-2.67, 2.67]
**Subtotal (95% CI)**			**56**			**60**	**100.0%**	**-0.23 [-1.51, 1.05]**

Heterogeneity: Tau² = 0.00; Chi² = 0.04, df = 1 (P = 0.85); I² = 0%
Test for overall effect: Z = 0.35 (P = 0.72)

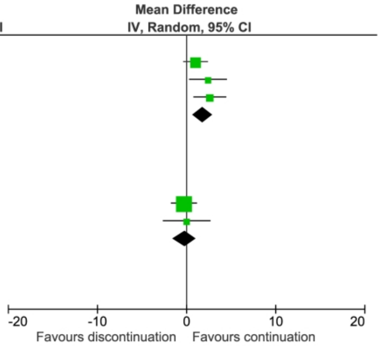

Figure 2. Forest plots of the Q-max when discontinuing or continuing alpha-blockers

PVR - 3 months

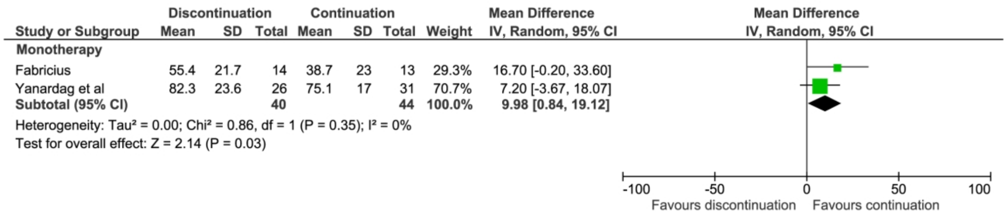

Figure 3. Forest plot of the PVR 3 months after discontinuing or continuing alpha-blockers

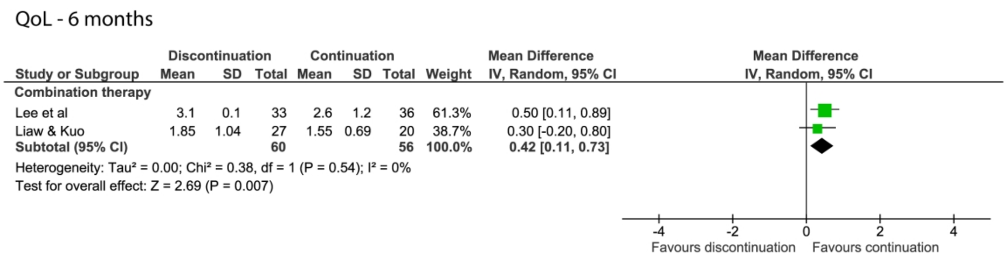

Figure 4. Forest plot of the QoL score 6 months after discontinuing or continuing alpha-blockers

Adverse events

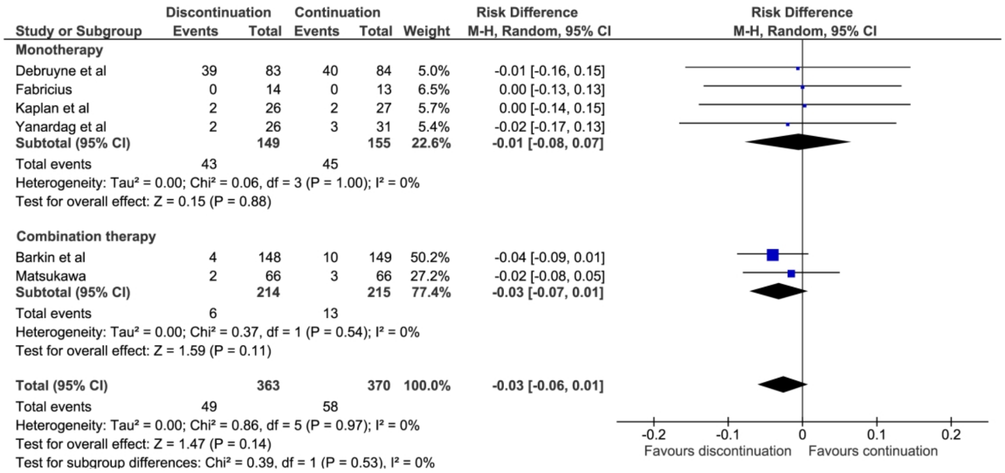

Figure 5. Forest plot of AEs after discontinuing or continuing alpha-blockers

Supplementary File 1. Search terms

Search terms

Adrenergic alpha blockers [MeSH] AND (LUTS OR BPH OR lower urinary tract symptoms OR benign prostate hypertrophy OR benign prostate enlargement) AND (discontinu* OR interrup* OR cessa* OR stop* OR withdra* OR intermit*).

For peer review only

Supplementary File 2. Flow diagram of inclusion and exclusion of studies

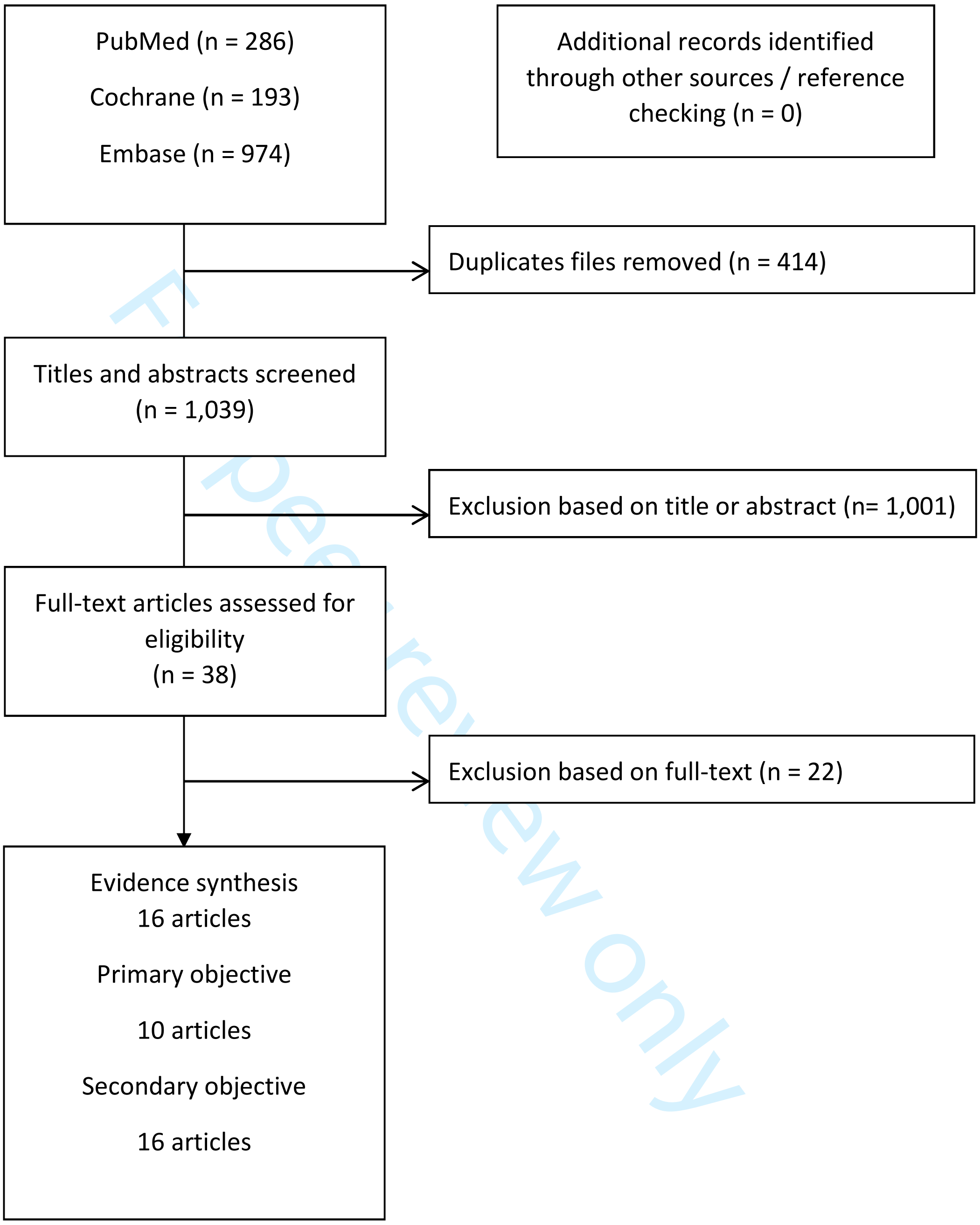

For peer review only

Supplementary File 3. Imputation of SD data from a previous meta-analysis

Tool	Ref.	Main author	Groups	N	SD	Pooled SD by study	Pooled SD overall
**IPSS**		**N = 12 studies**					
	49	Chapple	no data				
	73	Djavan	no data				
	147	Kirby	1	250	5.8	6.3	
			2	239	6.2		
			3	265	6.2		
			4	253	6.9		
	160	Lepor	no data				
	163	Lepor	no data				
	191	McConnell	no data				
	201	Mohanty	placebo	33	4	4.2	
			AB	36	4.4		
	208	Narayan	no data				
	254	Roehrborn	no data				
	255	Roehrborn	no data				
	262	Roehrborn	no data				
	305	van Kerrebroeck	N/A				
							**6.2**
**Q-max**		**N = 21 studies**					
	6	Abrams	N/A				
	33	Brawer	no data				
	50	chapple	no data				
	51	chapple	meta-analysis				
	53	christensen	N/A				
	79	elhilali	no data				
	101	gillenwater	no data				
	138	Kawabe	N/A				
	147	kirby	1	250	4.9		
			2	239	4.7	4.7	
			3	265	5.1		
			4	253	4.2		
	160	Lepor	no data				
	161	Lepor	no data				
	163	Lepor	no data				
	169	Lloyd	1	20	3.6		
			2	19	3.5	3.5	
			3	19	3.9		
			4	22	2.8		
	184	Martorana	N/A				
	201	Mohanty	placebo	33	2.6		
			AB	36	4.6	3.8	

For peer review only

For peer review only - http://bmjopen.bmj.com/site/about/guidelines.xhtml

208	Narayan	no data				
254	Roehrborn	no data				
255	Roehrborn	no data				
262	Roehrborn	no data				
269	Schulman	N/A				
305	van Kerrebroeck	N/A				
						**4.6**
**QoL**	**N = 5**					
49	chapple	placebo	350	1		
		ocas 0.4	354	1.1	1.1	
		mr 0.4	699	1.1		
		ocas 0.8	706	1.1		
160	Lepor	no data				
254	Roehrborn	no data				
255	Roehrborn	placebo	763	1.1		
		alfuzosin	759	1.1	1.1	
305	van Kerrebroeck	N/A				
						**1.1**

In total, 12 studies assessed the IPSS, 21 assessed Q-max, and 5 assessed the IPSS QoL domain. Only studies that reported an SD at follow-up were included for the IPSS and Q-max data. No follow-up SDs were available for the QoL data, so baseline SDs were used. The SDs were imputed based on Table 6-35 from the NICE guideline on the management of LUTS [ref. 3].

Abbreviations: IPSS, International Prostate Symptom Score; N/A, article not accessible; Q-max, Peak urine flow rate; QoL, Quality of life; SD, standard deviation.

For peer review only

Supplementary File 4. Results of the risk of bias assessment

	Random sequence generation (selection bias)	Allocation concealment (selection bias)	Blinding of participants and personnel (performance bias)	Blinding of outcome assessment (detection bias)	Incomplete outcome data (attrition bias)	Selective reporting (reporting bias)	Other bias
Barkin et al	?	?	+	+	+	+	+
Debruyne et al	+	+	+	+	+	+	+
Fabricius	?	?	+	+	+	+	+
Gerber et al	−	−	−	−	−	+	+
Kaplan et al	−	−	−	−	+	+	+
Lee et al	+	+	−	−	−	+	+
Liaw & Kuo	−	?	−	−	?	+	?
Lin et al	+	?	−	−	+	+	+
Matsukawa	+	+	−	−	+	+	+
Yanardag et al	?	?	−	?	+	+	+

view only

PRISMA 2009 Checklist

Section/topic	#	Checklist item	Reported on page #
**TITLE**			
Title	1	Identify the report as a systematic review, meta-analysis, or both.	1
**ABSTRACT**			
Structured summary	2	Provide a structured summary including, as applicable: background; objectives; data sources; study eligibility criteria, participants, and interventions; study appraisal and synthesis methods; results; limitations; conclusions and implications of key findings; systematic review registration number.	2
**INTRODUCTION**			
Rationale	3	Describe the rationale for the review in the context of what is already known.	4
Objectives	4	Provide an explicit statement of questions being addressed with reference to participants, interventions, comparisons, outcomes, and study design (PICOS).	4
**METHODS**			
Protocol and registration	5	Indicate if a review protocol exists, if and where it can be accessed (e.g., Web address), and, if available, provide registration information including registration number.	4
Eligibility criteria	6	Specify study characteristics (e.g., PICOS, length of follow-up) and report characteristics (e.g., years considered, language, publication status) used as criteria for eligibility, giving rationale.	5
Information sources	7	Describe all information sources (e.g., databases with dates of coverage, contact with study authors to identify additional studies) in the search and date last searched.	5
Search	8	Present full electronic search strategy for at least one database, including any limits used, such that it could be repeated.	Supplement
Study selection	9	State the process for selecting studies (i.e., screening, eligibility, included in systematic review, and, if applicable, included in the meta-analysis).	5-6
Data collection process	10	Describe method of data extraction from reports (e.g., piloted forms, independently, in duplicate) and any processes for obtaining and confirming data from investigators.	5-6
Data items	11	List and define all variables for which data were sought (e.g., PICOS, funding sources) and any assumptions and simplifications made.	5-6
Risk of bias in individual studies	12	Describe methods used for assessing risk of bias of individual studies (including specification of whether this was done at the study or outcome level), and how this information is to be used in any data synthesis.	6
Summary measures	13	State the principal summary measures (e.g., risk ratio, difference in means).	6
Synthesis of results	14	Describe the methods of handling data and combining results of studies, if done, including measures of consistency (e.g., I^2) for each meta-analysis.	6

For peer review only - http://bmjopen.bmj.com/site/about/guidelines.xhtml

PRISMA 2009 Checklist

Section/topic	#	Checklist item	Reported on page #
Risk of bias across studies	15	Specify any assessment of risk of bias that may affect the cumulative evidence (e.g., publication bias, selective reporting within studies).	6
Additional analyses	16	Describe methods of additional analyses (e.g., sensitivity or subgroup analyses, meta-regression), if done, indicating which were pre-specified.	NA
**RESULTS**			
Study selection	17	Give numbers of studies screened, assessed for eligibility, and included in the review, with reasons for exclusions at each stage, ideally with a flow diagram.	7
Study characteristics	18	For each study, present characteristics for which data were extracted (e.g., study size, PICOS, follow-up period) and provide the citations.	7 + supplement
Risk of bias within studies	19	Present data on risk of bias of each study and, if available, any outcome level assessment (see item 12).	7+ Supplement
Results of individual studies	20	For all outcomes considered (benefits or harms), present, for each study: (a) simple summary data for each intervention group (b) effect estimates and confidence intervals, ideally with a forest plot.	Figures
Synthesis of results	21	Present results of each meta-analysis done, including confidence intervals and measures of consistency.	8-12 + supplement
Risk of bias across studies	22	Present results of any assessment of risk of bias across studies (see Item 15).	7-9+supplement
Additional analysis	23	Give results of additional analyses, if done (e.g., sensitivity or subgroup analyses, meta-regression [see Item 16]).	NA
**DISCUSSION**			
Summary of evidence	24	Summarize the main findings including the strength of evidence for each main outcome; consider their relevance to key groups (e.g., healthcare providers, users, and policy makers).	12-13
Limitations	25	Discuss limitations at study and outcome level (e.g., risk of bias), and at review-level (e.g., incomplete retrieval of identified research, reporting bias).	13-14
Conclusions	26	Provide a general interpretation of the results in the context of other evidence, and implications for future research.	14
**FUNDING**			
Funding	27	Describe sources of funding for the systematic review and other support (e.g., supply of data); role of funders for the systematic review.	3

From: Moher D, Liberati A, Tetzlaff J, Altman DG, The PRISMA Group (2009). Preferred Reporting Items for Systematic Reviews and Meta-Analyses: The PRISMA Statement. PLoS Med 6(7): e1000097.

For peer review only - http://bmjopen.bmj.com/site/about/guidelines.xhtml

PRISMA 2009 Checklist

doi:10.1371/journal.pmed1000097 For more information, visit: **www.prisma-statement.org**. Page 2 of 2

For peer review only

BMJ Open

Discontinuation of alpha-blocker therapy in men with lower urinary tract symptoms: A systematic review and meta-analysis

Journal:	*BMJ Open*
Manuscript ID	bmjopen-2019-030405.R1
Article Type:	Original research
Date Submitted by the Author:	05-Oct-2019
Complete List of Authors:	van der Worp, Henk; University of Groningen, University medical center groningen, Department of General Practice and Elderly Care Medicine<br>Jellema, Petra; University of Groningen, University medical center groningen, Department of General Practice and Elderly Care Medicine<br>Hordijk, Ilse; Isala Hospitals, urology<br>Lisman-van Leeuwen, Yvonne; University of Groningen, University medical center groningen, Department of General Practice and Elderly Care Medicine<br>Korteschiel, Lisa; Isala Hospitals, urology<br>Steffens, Martijn ; Isala Hospitals, urology<br>Blanker, Marco; University of Groningen, University medical center groningen, Department of General Practice and Elderly Care Medicine
**Primary Subject Heading**:	Urology
Secondary Subject Heading:	General practice / Family practice
Keywords:	alpha-blockers, discontinuation, lower urinary tract symptoms, meta-analysis

SCHOLARONE™ Manuscripts

Discontinuation of alpha-blocker therapy in men with lower urinary tract symptoms: A systematic review and meta-analysis

Henk van der Worp, PhD, senior-researcher (0000-0001-5545-4155) [1], Petra Jellema, PhD, senior-researcher and epidemiologist [1], Ilse Hordijk, MD [3], Yvonne Lisman-van Leeuwen, PhD, epidemiologist [1], Lisa Korteschiel, MD [2], Martijn G Steffens, MD PhD, urologist [2], Marco H Blanker, MD PhD, GP and epidemiologist [1]

1. Department of General Practice and Elderly Medicine, University of Groningen, University Medical Centre Groningen, Groningen, the Netherlands
2. Department of Urology, Isala Clinics Zwolle, Zwolle, the Netherlands

Correspondence to:

Dr. Henk van der Worp

Department of General Practice and Elderly Medicine, University of Groningen, University Medical Centre Groningen, FA21, PO Box 196, 9700AD, Groningen, the Netherlands

h.van.der.worp@umcg.nl

+31 503615020

Word count: 3499

Keywords: alpha-blockers; discontinuation; lower urinary tract symptoms; meta-analysis

For peer review only - http://bmjopen.bmj.com/site/about/guidelines.xhtml

ABSTRACT

Objectives: We aimed to synthesize the available data for the effect of stopping alpha-blocker therapy among men with lower urinary tract symptoms. The focus was on symptom, uroflowmetry, and quality of life outcomes, but we also reviewed the adverse events and the number of patients who restarted therapy.

Eligibility criteria: We selected studies regardless of study design in which men were treated with an alpha-blocker for at least 3 months and in which the effects of alpha-blocker discontinuation were subsequently studied. Only controlled trials were used for the primary objective.

Information Sources: We searched MEDLINE/PubMed, EMBASE/Ovid, and The Cochrane Central Register of Controlled Trials from inception to May 2018

Risk of bias: Two reviewers independently assessed the risk of bias for the controlled studies only, using the Cochrane Collaboration's tool for assessing risk of bias.

Included studies: We identified ten studies (1,081 participants) assessing the primary objective. Six studies (733 participants) assessed differences in adverse events between continuation and discontinuation and six studies (501 participants) reported the numbers of subjects that restarted treatment after discontinuation. No studies in primary care were identified.

Synthesis of the results: After discontinuing monotherapy, symptom scores increased and peak flow rates decreased at 3 and 6 months, but not at 12 months; however, neither parameter changed when alpha-blockers were stopped during combination therapy. Small differences in post-void residual volumes and quality of life scores were considered clinically irrelevant. We also found that 0%–49% of patients restarted after stopping alpha-blocker therapy and that adverse events did not increase with discontinuation.

Description of the effect: Discontinuing alpha-blocker monotherapy leads to a worsening

For peer review only - http://bmjopen.bmj.com/site/about/guidelines.xhtml

compared with continuing therapy. Discontinuing the alpha-blocker after combination therapy had no significant effects on outcomes in either the short or long term.

Interpretation: We conclude that discontinuation may be appropriate for the frail, elderly, or those with concomitant illness or polypharmacy. However, studies in primary care are lacking.

Registration: PROSPERO database (CRD42016032648)

https://www.crd.york.ac.uk/prospero/display_record.php?RecordID=32648

Strengths and limitations of the study:

- This is the first systematic review that synthesizes the literature concerning alpha-blocker discontinuation.
- The review was conducted in accordance with the PRISMA-guidelines.
- The number of studies that could be included was limited and the risk of bias was high for most outcomes preventing the drawing of firm conclusions.

Role of funding sources

This research received no specific grant from any funding agency in the public, commercial or not-for-profit sectors

Competing interest declaration

All authors have completed the ICMJE uniform disclosure form at www.icmje.org/coi_disclosure.pdf and declare: no support from any organisation for the submitted work; no financial relationships with any organisations that might have an interest in the submitted work in the previous three years; no other relationships or activities that could appear to have influenced the submitted work.

For peer review only - http://bmjopen.bmj.com/site/about/guidelines.xhtml

INTRODUCTION

Alpha-blockers are the first-choice treatment for men with moderate-to-severe lower urinary tract symptoms (LUTS) because of their proven, but small, superiority over placebo,[1-3] but their use can associated with dizziness, orthostatic hypotension, and increased fall risk.[3, 4] This may be especially problematic in the elderly, who often have polypharmacy and multi-morbidity. Given the natural course of LUTS, with 30% of patients showing improvement over time,[5] it may be appropriate to consider discontinuation of alpha-blocker therapy, especially in the elderly. There are no clear data on the effect of this approach but the guideline on male LUTS for Dutch general practitioners advises that alpha-blocker therapy be discontinued after 3–6 months, followed by symptom review.[2] By contrast, guidelines followed by urologists do not advocate routine discontinuation,[1, 6] though the European Association of Urology (EAU) do mention that alpha-blocker discontinuation may be considered after 6 months in the context of combination therapy.[1]

A number of researchers have studied the effects of discontinuing alpha-blockers, but to date, there has been no synthesis of this literature.

We performed a systematic review and meta-analysis to obtain data about the effect of discontinuing alpha-blockers on male LUTS. Our primary objective was to compare the effects of discontinuing therapy with those of continuing therapy. Secondary objectives were (1) to determine the proportion of men who restart alpha-blocker therapy and (2) to determine the possible adverse effects of both discontinuation and continuation.

METHODS

We completed this review according to the Preferred Reporting Items for Systematic Reviews and Meta-Analyses (PRISMA) and registered the protocol in the PROSPERO database (CRD42016032648) http://www.crd.york.ac.uk/PROSPERO/display_record.php?ID=CRD42016032648.

For peer review only - http://bmjopen.bmj.com/site/about/guidelines.xhtml

Selection criteria

We selected studies in which men were treated with an alpha-blocker for at least 3 months and in which the effects of alpha-blocker discontinuation were subsequently studied. For the primary objective, only randomized controlled trials (RCTs, including quasi-randomized trials) and non-randomized trials (NRTs) that compared alpha-blocker discontinuation to continuation were selected. For the secondary objectives, we also included uncontrolled studies. At all stages, we excluded studies written in languages other than Dutch, English, French, or German.

Outcome measures

The following outcomes were used for the primary objective: symptom scores, such as the International Prostate Symptom Score (IPSS); urinary flow rates; post-void residual urine volume (PVR); and quality of life (QoL). For the secondary objectives, we calculated the percentage of patients who restarted alpha-blocker therapy and the numbers of adverse events (AEs) in the continuation and discontinuation groups.

Search methods for identification of studies

We searched MEDLINE/PubMed, EMBASE/Ovid, and The Cochrane Central Register of Controlled Trials using search terms covering LUTS, alpha-blockers, and discontinuation (see Supplementary File 1 for detailed information). We ran the searches in January 2016 and updated them in July 2017 and May 2018. The reference lists of relevant articles were also screened to identify additional eligible studies. All duplicate files were removed before the titles and abstracts of the remaining records were independently screened by three reviewers [IH, LK, MB] and classified as "inclusion," "exclusion," or "uncertain." Next, the same reviewers independently applied the selection criteria to the full-text papers of all records classified into the inclusion or uncertain groups, and decided whether to include or exclude the research. Discrepancies in the selection procedure were resolved by consensus.

Data extraction process

Two authors [HW, IH] independently performed data extraction using standardized forms.

For peer review only - http://bmjopen.bmj.com/site/about/guidelines.xhtml

We extracted the following data: 1) the participant characteristics; 2) the interventions used; 3) the primary and secondary outcomes, as well as the timing of the outcome assessment; and 4) the study design. If possible, we extracted data by allocated intervention to allow an intention-to-treat analysis. Discrepancies were resolved by re-examination and discussion of the full-text papers or by consultation with a third author [MB].

Risk of bias

Two reviewers [HW, YL] independently assessed the risk of bias using the Cochrane Collaboration's tool for assessing risk of bias.[7] This tool includes six domains, as follows: selection bias, performance bias, detection bias, attrition bias, reporting bias, and other bias. Only RCTs and NRCTs were assessed, because these were used for the primary objective. Discrepancies in the risk of bias assessment were resolved by consensus or arbitration with a third party [MB]. Risk of bias was described for the five domains (selection, performance, attrition, reporting, and other) and summarized across studies and outcomes.[7]To ascertain graphically the existence of publication bias, the construction of funnel plots was planned in case at least 10 studies were included.

Data analysis

Data were analyzed using Review Manager, Version 5.3.[8] We calculated the risk difference and 95% confidence intervals (CIs) for dichotomous variables (inverse-variance method) and the mean differences (MD) with 95% CIs for continuous variables (Mantel-Haenszel method). For AEs, we also calculated the rate of AEs per 1,000 patient-days, based on the sample sizes and follow-up times.

Clinical heterogeneity was assessed by checking the characteristics of participants and interventions. Statistical heterogeneity was assessed by visual inspection of the forest plots and of the results of statistical testing for heterogeneity (I^2 statistic). We pooled data if we identified two or more studies with an I^2 of <40%,[9] using a random effects model. Data from both RCTs and NRCTs were pooled. Synthesizing and pooling were done separately for monotherapy and combination therapy. If the data for pooling were only presented in figures (e.g., standard deviations), it was

For peer review only - http://bmjopen.bmj.com/site/about/guidelines.xhtml

extracted from those figures. If data was not present at all in the article, we contacted the authors if the article had been published in the past 10 years. If data could not be obtained in this way, we imputed data from a previous meta-analysis on the efficacy of alpha-blockers,[3] as described in the Cochrane Handbook.[9, 10]

The following cut-off values were used to define the minimal clinical important difference (MCID): 2.7 points for IPSS,[27] 2mL/s for Q-max,[3] and 0.5 points for the IPSS-QoL scores.[3] The MCID is the smallest change in a treatment outcome that an individual patient would identify as important and which would indicate a change in the patient's management.

Patient and public involvement

This study was performed without patient involvement. Patients were not invited to comment on the study design and were not consulted to interpret the results. Patients were not invited to contribute to the writing or editing of this document for readability or accuracy.

RESULTS

The searches yielded 1,039 publications (Supplementary File 2), of which 16 with a total of 1,823 participants were included (Table 1). All included studies were performed in secondary or tertiary care. Nine studies (772 participants) reported discontinuing alpha-blocker monotherapy: two double-blind RCTs,[11, 12] two open-label RCTs,[13, 14] one NRCT,[15] and four uncontrolled studies.[16-19] Six studies (980 participants) reported discontinuing alpha-blockers used in combination therapy: one double-blind RCT,[20] four open-label RCTs,[21-24] and one uncontrolled study.[25] Finally, one uncontrolled study (N = 71) reported discontinuing both alpha-blocker monotherapy and combination therapy.[26]

Two of the included studies randomized patients into three groups: a discontinuation group, a continuation group, and a third group that continued with alternate-day use of alpha-blockers.[13, 14] We only used the data from the discontinuation and continuation groups.

For peer review only - http://bmjopen.bmj.com/site/about/guidelines.xhtml

For peer review only

Table 1. Characteristics of the controlled and uncontrolled studies of monotherapy and combination therapy

Authors	Design	Type of AB	Daily dose	AB Use (Before Stopping)	No. Patients (Stop Phase)	Age (Mean)	IPSS at Baseline (Mean)	Measured Outcomes Primary	Measured Outcomes Secondary	Follow-Up (Months)
**Controlled trials**										
Monotherapy										
Fabricius et al. 1990 [12]	Double-blind RCT	TER	10 mg	24 wk	27	68	-	Q-max, Q-avg, PVR,	AEs	3
Debruyne et al. 1996 [11]	Double-blind RCT	TER	5 mg/10 mg	26 wk	167	63.6	19.1	IPSS, Q-max, QoL	AEs	6
Gerber et al. 1997 [15]	NRT	DOX	4 mg	3 mo	37	65	C=20.9; DC=21.5	IPSS	Restart	12
Kaplan et al. 1998 [14]	Open-label RCT (quasi-randomized)	ALF	7.5 mg	3 mo	53	60.5	15.6	IPSS, Q-max	AEs	3/6
Yanardag et al. 2005 [13]	Open-label RCT	TAM	0.4 mg	3 mo	57	61.3	12.3	IPSS, Q-max, Q-avg, PVR	AEs	3/6
Combination therapy										
Barkin et al. 2003 [20]	Double-blind RCT	TAM	0.4 mg	24 wk	277	C=67.6; DC=66.9	C=16.4; DC=16.5	IPSS, QoL	AEs	3
Liaw & Kuo 2006 [24]	Open-label RCT (quasi-randomized)	TAM	0.2 – 0.4 mg	1 yr	47	C=70.7; DC=72.1	15.6	IPSS, Q-max, QoL	Restart	3/6/12
Lee et al. 2012 [23]	Open-label RCT	TAM	0.2 mg	48 wk	69	68	15.3	IPSS, Q-max, QoL	Restart, AEs	6
Lin et al. 2014 [22]	Open-label RCT	DOX	4 mg	2 yrs	230	75	C=13.1; DC=15.6	IPSS, Q-max, PVR	Restart	12
Matsukawa et al. 2017 [21]	Open-label RCT	SIL	8 mg	12 mo	117	C=70.1; DC=69.1	C=17.4; DC=17.2	IPSS, Q-max, PVR, QoL	AEs	12
**Uncontrolled studies**										
Monotherapy										
Kobayashi et al. 2006 [17]	CS	TAM	0.2 mg	28.5 ± 26.8 mo	33	70.4	16.3		Restart	6
Yokoyama et al. 2007 [19]	CS	NAF/ TAM /URA	25-50 mg/0.2 mg/30 mg	2–200 mo	60	70 (median)	15.9		Restart	12
Nickel et al. 2008 [18]	CS	ALF/DOX/ TAM	No data	9 mo	220	66.1 (total sample)	19.9		Restart	9

For peer review only - http://bmjopen.bmj.com/site/about/guidelines.xhtml

		/TER								
Chung et al. 2013 [16]	CS	ALF	10 mg	12 wk	58	68.6 (total sample)	16.7 (total sample)		Restart	6
Combination therapy										
Baldwin et al. 2001 [25]	CS	DOX	2-8 mg	3–12 mo	240	66 (total sample)	Range: 20–33 (total study sample)		Restart	1
*Both**										
Kuo 1998 [26]	CS	DIB	20 mg	6 mo	ABM=71; ABC=65	ABM=66.3; ABC=66.8	ABM=21.2; ABC=22.5		Restart	1

AB, alpha-blocker; ABC, alpha-blocker combination treatment; ABM, alpha-blocker monotherapy; AEs, adverse events; ALF = Alfuzosin; C, continuation group; CS, cohort study; DC, discontinuation group; DIB = Dibenyline; DOX = Doxazosin; NAF = Naftopidil; NRCT, non-randomized controlled trial; ns, not stated; PVR, post voided residual urine volume; Q-avg, average urine flow rate; Q-max, peak urine flow rate; SIL = Silodosin; TAM = Tamsulosin; TER = Terazosin; URA = Urapidil.

* both monotherapy and combination therapy discontinuation

For peer review only

For peer review only

For peer review only - http://bmjopen.bmj.com/site/about/guidelines.xhtml

In another three studies, the data required for pooling were missing.[11, 12, 20] Because these studies had been published over 15 years previously, no efforts were made to contact the authors. For one of the studies, means and standard deviations could be obtained from the figures.[12] For the other two studies,[11, 20] the standard deviations were missing and were imputed from the results of a previous meta-analysis (see Supplementary File 3).[3] No studies were excluded form pooling based on statistical heterogeneity.

Risk of bias

Most of the included studies had risks of bias (Supplementary File 4). The most common were lack of blinding and randomization, with only three out of ten studies having a “low risk” for these items. There was no evidence of reporting bias in any of the included studies. The summary of bias by study indicated that only one study had low risk of bias,[11] while two studies had unclear risks of bias,[12, 20] and the remaining studies had high risks of bias.[13-15, 21-24] As a result, risk of bias was high for all but one outcome.

We did not construct funnel plots to ascertain the existence of publication bias graphically, as the number of included studies in each meta-analysis was less than 10.

Effects of alpha-blocker discontinuation

Five studies of monotherapy (n = 341) [11, 12, 14, 15] and five studies of combination therapy (n=740) [20-24] were used for the primary research objective. Only three provided data on the number of patients who did not comply with the intervention and who restarted alpha-blocker use after discontinuation. Two of these provided a per protocol analysis excluding those patients [23, 24] and the third provided an intention-to-treat analysis for categorized variables, with a per protocol analysis for the raw outcomes,[22] effectively precluding an intention-to-treat analysis. Because all included studies reported outcomes at 3, 6, or 12 months, we compared outcomes at these time points.

Symptom scores

For peer review only - http://bmjopen.bmj.com/site/about/guidelines.xhtml

All but one study [12] assessed symptoms with the IPSS questionnaire, or its predecessor the AUA symptom score (n = 1,054).

By 3 months after discontinuing alpha-blocker monotherapy, symptoms increased in the discontinuation group compared with the continuation group (MD = 4.17; 95% CI 2.91 to 5.43),[13, 14] whereas there was no difference between the continuation and discontinuation groups in the studies of combination therapy (MD = 0.97; 95% CI -0.32 to 2.27, Figure 1A).[20, 24]

After 6 months, two RCTs and one NRCT on monotherapy found a significant worsening of symptoms (differences varying from 2.0 to 5.8 points) in subjects that discontinued alpha-blockers.[11, 13, 14] No difference was found for studies on combination therapy after 6 months (MD = 0.56; 95% CI -1.57;2.69, Figure 1B).[23, 24]

After 12 months, the one study that looked at discontinuing monotherapy found a non-significant difference of 1.2 points between groups.[15] No differences were found in two open-label RCTs that looked at alpha-blocker discontinuation after combination therapy.[21, 22] Another NRCT presented data for both groups, but did not make a direct statistical comparison between groups.[24] Data could not be pooled (I^2=61%).

Peak urine flow rate

Nine studies (804 patients) assessed peak urine flow rate (Q-max): five studies for monotherapy,[11-15] and four studies for combination therapy.[21-24]

After 3 months, Q-max reduced by 2.59 mL/s (95% CI 1.40 to 3.77, Figure 2A) in those who discontinued alpha-blocker monotherapy compared with those who continued therapy.[12-14] A single study on discontinuing combination therapy found that there was an increase of 1.4 mL/s in those who discontinued compared to those who continued therapy, but the researchers did not perform a statistical comparison.[24]

After 6 months, a reduction was again found in the Q-max after discontinuing monotherapy (MD = 1.79; 95% CI 0.73 to 2.86),[11, 13, 14] but no difference was found after discontinuing in the context of combination therapy (MD -0.23; 95% CI -1.51 to 1.05, Figure 2B).[23, 24]

For peer review only - http://bmjopen.bmj.com/site/about/guidelines.xhtml

After 12 months, no differences were found in an NRCT reporting on the effects of discontinuing monotherapy.[15] Three studies assessed Q-max 12 months after combination therapy. Among these, two open-label RCTs found no difference between groups:[21, 22] one study found a difference of 0.1 mL/s in favor of the continuation group and the other found that 7% fewer patients in the discontinuation group had a reduction in Q-max of >2 mL/s compared with the continuation group. Again, the NRCT on combination therapy showed an increase of 2.5 mL/s after alpha-blocker discontinuation, which was not seen in the group that continued alpha-blockers, but differences were not tested.[24] Data could not be pooled (I^2 = 68%).

Average urine flow rate (Q-avg)

Data from two RCTs on monotherapy (84 patients) could not be pooled (I^2 = 70%).[12, 13] After 3 months, one RCT reported a reduction of 2.2 mL/s in subjects who discontinued therapy compared with those who continued therapy,[12] whereas no statistical testing of the difference of 0.6 mL/s between groups was performed in the other RCT.[13]

After 6 months, this second study found a difference of 0.9 mL/s between groups in favor of continuing monotherapy.[13]

Post voided residual urine volume (PVR)

PVR volume was measured in five studies (n = 468), with three measuring it after discontinuing monotherapy [12, 13, 15] and two measuring it after discontinuing the alpha-blocker in combination therapy.[21, 22]

After 3 months, discontinuing monotherapy resulted in a PVR volume increase of 9.98 mL (95% CI 0.84 to 19.12, Figure 3).[12, 13]

After 6 months, an open-label trial on discontinuing monotherapy (57 participants) also found a statistically significant difference (14.1 mL) in favor of continuing therapy.[13]

At 12 months after discontinuing monotherapy, an NRCT did not find a significant difference between groups.[15] Two open-label RCTs on discontinuing combination therapy did report non-

For peer review only - http://bmjopen.bmj.com/site/about/guidelines.xhtml

significant differences: one showed a 2 mL difference between groups,[21] and the other showed that 8% more patients in the discontinuation group reported a PVR increase of >50%.[22]

Quality of life

All five studies (one of monotherapy and four of combination therapy; 677 patients) that assessed QoL used the IPSS QoL sub-score.

After 3 months, one study of combination therapy found no difference between groups (0 points).[20] In another NRCT of combination therapy, a difference of 0.4 points was reported in favor of the group that discontinued therapy, but this was not statistically tested.[24]

After 6 months, one study on monotherapy found a statistically significant difference of 0.2 points in favor of those who continued alpha-blocker therapy.[11] This was the only outcome with a low risk of bias. A difference was found for the pooled studies of combination therapy (MD = 0.42; 95% CI 0.11–0.73, Figure 4).[23, 24]

After 12 months, no differences in QoL scores (only 0.1 points in favor of discontinuation) were found in an RCT of 117 participants receiving combination therapy.[21] Another NRCT of patients receiving combination therapy found a difference of 0.4 in favor of continuation, but did not compare groups statistically.[24] Data could not be pooled (I^2 = 60%).

Restart of prior treatment and AEs

Patients restarting treatment

Six studies (501 patients) reported data on restarting alpha-blockers after discontinuation.[15, 16, 19, 22-24] In three of these (187 patients), 7%–49% of subjects restarted alpha-blockers after 6 months.[16, 19, 23] One of these,[19] together with another three studies (374 patients), [15, 22, 24] described that 0%–33% of subjects had restarted alpha-blockers at 12 months. The highest (49%) and lowest percentages (0%) restarting therapy were found in studies of monotherapy.[15, 16] However, four of the included studies explicitly advised subjects to restart alpha-blocker use if their PVR was >100 mL,[16, 19] or if symptoms worsened.[22, 24] These studies

For peer review only - http://bmjopen.bmj.com/site/about/guidelines.xhtml

reported the highest restart rates.

Three other studies provided indirect information about restarting alpha-blocker use.[17, 25, 26] Two of these reported on successful discontinuation, defined as no increase in symptoms and no request for continuation of treatment. After 6 months, one indicated success among 69% of those receiving monotherapy.[17] Another study reported success rates of 13%–87% one month after discontinuing combination therapy, with percentages increasing as the duration of alpha-blocker use increased (ranging from 3 – 12 months).[25] Discontinuation was successful in 13%–20% of subjects who used alpha-blockers for 3 months and in 84%–87% of subjects who used them for 12 months. A third study stated that most patients whose symptoms worsened after discontinuation wished to restart their medication rather than undergo surgery.[26]

Adverse events

Nine studies provided no data on AEs during discontinuation, or if they did, provided data without a clear indication of the treatment group.[23] Another study only reported AEs during follow-up for those who discontinued alpha-blockers.[18] The six remaining studies reported 49 AEs in 363 patients who discontinued alpha-blockers and 58 AEs in 370 patients who continued to use alpha-blockers.[11-14, 20, 21] The AE rates in patients who discontinued or continued alpha-blockers were 0.13 and 0.15 per 1,000 patient-days, respectively. The pooled data showed no risk difference for AEs when discontinuing or continuing either monotherapy (risk difference = -0.01; 95% CI -0.08 to 0.07) or combination therapy (risk difference = -0.03; 95% CI -0.07 to 0.01, Figure 5).

Respiratory tract infection and urinary retention were the two most common AEs after discontinuing alpha-blockers (11 studies in total), being reported in 1%–4% of patients [11, 20] and in 1%–3% of patients,[11, 21] respectively. The incidence of these AEs did not differ between groups.

DISCUSSION

The results of this systematic review indicate that discontinuing alpha-blocker monotherapy leads to a worsening of clinical symptoms and a decrease of urinary flow rates in the short-term (3–6

For peer review only - http://bmjopen.bmj.com/site/about/guidelines.xhtml

months) compared with continuing therapy. However, after one year, no differences were found in these or other outcomes. Discontinuing the alpha-blocker after combination therapy had no significant effects on outcomes in either the short or long term.

The worsening of symptoms over the short-term after stopping monotherapy was probably relevant to clinical practice. The reported differences in the IPSS between groups exceeded the minimal clinically important difference (MCID) of 2.7 points.[27] The difference in Q-max was also clinically relevant, exceeding the MCID of 2 mL/s after 3 months (between-group difference, 2.59 mL/s),[3] but not after 6 months (1.79 mL/s). One might argue about the relevance of this outcome for patient, as men will not be able to notice a difference in flow rate at these values. The difference of 0.42 points in the QoL scores at 6 months after discontinuing combination therapy remained below the MCID of 0.5 points.[3] Although no MCID was available for PVR, we do not think that the reported mean difference of 10 mL after 3 months was clinically relevant.

The worsening of symptoms noted by 3–6 months after discontinuing monotherapy was larger than the reported improvement of symptoms after initiating therapy, which was reported to be 2.55 points (95% CI, 1.92–3.17) based on 12 RCTs with a total of 9,335 participants.[3] The magnitude of change in the present review may have been influenced by the lack of blinding of both patients and assessors in many of the studies, which will have favored the continuation groups. Men in these studies who had no clear symptom improvements are likely to have dropped out before the discontinuation phase, so the participants subsequently included in the discontinuation trials will generally have had larger treatment effects and larger changes after discontinuation. The outcomes after 12 months relied on data from a single study on discontinuing doxazosin (not a controlled-release version),[15] which has a lower efficacy than other alpha-blockers. This might explain the lack of any meaningful long-term impact.

Among patients receiving combination therapy, outcomes were not significantly different between those discontinuing and continuing alpha-blockers. Although 5-alpha-reductase inhibitors have no significant impact on LUTS severity after treatment initiation,[28, 29] their continuation

For peer review only - http://bmjopen.bmj.com/site/about/guidelines.xhtml

seems to be protective against symptom worsening after discontinuing alpha-blockers.

The results for restarting a discontinued alpha-blocker were heterogeneous, ranging widely from 0% to 49%. These conflicting findings can be explained by the differences in instructions given to patients in these studies. Indeed, participants in some studies received explicit instructions about when to restart therapy, whereas in other studies, no instructions were given. Also, subjects in cohort studies who volunteered to discontinue therapy may have had greater freedom to restart therapy than those participating in an RCT.

It was also shown that discontinuing alpha-blockers did not result in more AEs, including acute urinary retention.[30] Equally, continuation was not associated with more AEs, with neither dizziness nor orthostatic hypotension being more common.[4] This may be explained by subject drop-out due to AEs before entering the discontinuation phase. The number of patients reporting AEs in the included studies was, however, too small to draw meaningful conclusions regarding AEs.

Interpretation of our findings is hampered by some limitations. For example, the limited numbers of studies and large amount of statistical heterogeneity limited data pooling. Heterogeneity, especially on IPSS outcomes after 6 months could be explained by differences in alpha-blockers studied and baseline symptom severity differences ranging from 12 to 19 in the included studies. The limited number of RCTs also precluded sensitivity analyses, and subgroup analyses, that were planned in the original review protocol. Another limitation related to the limited number of studies is the reduction in statistical power. It has been shown that at least five studies have to be pooled to achieve a greater power than the original studies independently.[31] So, our results could also be subject to Type I error. In addition, some studies gave unclear data about treatment compliance or only presented per protocol analyses, which may have led to bias (e.g., drop-out due to severe complaints) and loss of generalizability. Another issue is that all studies were performed in secondary or tertiary care settings. This is important if we consider that in some countries, most men with LUTS are treated in primary care. The high risk of bias, which was noted for all but one outcome, also hampers the interpretation of our findings. Finally, two of the trials of

For peer review only - http://bmjopen.bmj.com/site/about/guidelines.xhtml

combination therapy compared discontinuing alpha-blockers and 5-alpha-reductase inhibitors, but the others compared discontinuing and continuing only the alpha-blocker.[22, 24]

No firm conclusions can be drawn from this review because of the low quality of the available evidence. Overall, the data suggest that there is a short-term clinical worsening of LUTS after discontinuing alpha-blocker monotherapy, as assessed by symptom scores and urinary flow rates, but that this does not increase the risk of a complicated symptom course.

Patients frequently discontinue alpha-blocker treatment in clinical practice. We have recently shown that men who continue to use alpha-blockers are typically unconcerned about stopping that therapy if advised to do so by a doctor.[32] The present review also provides evidence that the magnitude of symptom deterioration is limited, indicating that physicians can change their prescribing policy without risking harm. Indeed, the alternative approach may promote unnecessary polypharmacy, which is especially relevant in vulnerable groups. Active follow-up should then be used to monitor the need to restart alpha-blockers if symptoms worsen.

Our findings support the existing EAU guidance to consider discontinuing alpha-blockers in patients receiving combination therapy for 6 months.[1] Unfortunately, because the studies in this review were only performed in secondary care, we cannot give firm support for the recommendation of the Dutch GP guideline to review therapy after 3–6 months in primary care.[2] Symptom levels before treatment are generally lower in primary care, where conditions are typically less severe than in secondary care. Although the data from this review may be applicable to primary care, further efficacy studies and discontinuation trials are needed to assess the outcomes specific to this setting.

Acknowledgments

We thank Dr Robert Sykes (www.doctored.org.uk) for providing editorial services.

Author statement

For peer review only - http://bmjopen.bmj.com/site/about/guidelines.xhtml

The corresponding author attests that all listed authors meet authorship criteria and that no others meeting the criteria have been omitted.

MHB had the idea for the article. Acquisition of the data was done by IH, LK, HW and MHB. Analysis and interpretation of the data was done by HW, MHB, PJ, YL and MGS. HW and MHB wrote the manuscript. All authors critically reviewed the manuscript.

Data sharing statement

Data collected for this study will be available from the corresponding author upon request.

For peer review only

References

1 Gratzke C, Bachmann A, Descazeaud A, et al. EAU Guidelines on the Assessment of Non-neurogenic Male Lower Urinary Tract Symptoms including Benign Prostatic Obstruction, *Eur Urol* 2015;67:1099-109.

2 Blanker M, Klomp M, Donk M, et al. Summary of the NHG practice guideline 'Lower urinary tract symptoms in men', *Ned Tijdschr Geneeskd* 2013;157:A6178.

3 National Clinical Guideline Centre (NICE).
The management of lower urinary tract symptoms in men: NICE guideline. 2010;CG97(01/09).

4 Bird ST, Delaney JA, Brophy JM, et al. Tamsulosin treatment for benign prostatic hyperplasia and risk of severe hypotension in men aged 40-85 years in the United States: risk window analyses using between and within patient methodology, *BMJ* 2013;347:f6320.

5 Kok ET, Bohnen AM, Groeneveld FP, et al. Changes in disease specific and generic quality of life related to changes in lower urinary tract symptoms: the Krimpen study, *J Urol* 2005;174:1055-8.

6 McVary KT, Roehrborn CG, Avins AL, et al. Update on AUA guideline on the management of benign prostatic hyperplasia, *J Urol* 2011;185:1793-803.

7 Higgins JP, Altman DG, Gotzsche PC, et al. The Cochrane Collaboration's tool for assessing risk of bias in randomised trials, *BMJ* 2011;343:d5928.

8 Anonymous . Review Manager (RevMan) [Computer program]. Version 5.3. Copenhagen: The Nordic Cochrane Centre. 2014.

9 Anonymous . Cochrane Handbook for Systematic Reviews of Interventions Version 5.1.0 [updated March 2011].: The Cochrane Collaboration 2011.

10 Furukawa TA, Barbui C, Cipriani A, et al. Imputing missing standard deviations in meta-analyses can provide accurate results, *J Clin Epidemiol* 2006;59:7-10.

11 Debruyne FM, Witjes WP, Fitzpatrick J, et al. The international terazosin trial: a multicentre study of the long-term efficacy and safety of terazosin in the treatment of benign prostatic hyperplasia. The ITT Group, *Eur Urol* 1996;30:369-76.

12 Fabricius P G PG. Efficacy of once-a-day terazosin in benign prostatic hyperplasia: a randomized, double-blind placebo-controlled clinical trial. *The Prostate.Supplement* 1990;3:85-93.

13 Yanardag H, Goktas S, Kibar Y, et al. Intermittent tamsulosin therapy in men with lower urinary tract symptoms, *J Urol* 2005;173:155-7.

14 Kaplan SA, Reis RB, Cologna A, et al. Intermittent alpha-blocker therapy in the treatment of men with lower urinary tract symptoms, *Urology* 1998;52:12-6.

15 Gerber GS, Contreras BA, Zagaja GP, et al. Doxazosin in men with lower urinary tract symptoms: urodynamic evaluation at 15 months, *Urology* 1997;50:229-33.

For peer review only

For peer review only - http://bmjopen.bmj.com/site/about/guidelines.xhtml

16 Chung JH, Lee JY, Kang DH, et al. Evaluation of patient outcome after discontinuation of alfuzosin treatment for benign prostatic hyperplasia: a multicentre, prospective study, *Int J Clin Pract* 2013;67:870-5.

17 Kobayashi M, Tokue A, Morita T. Discontinuation of tamsulosin treatment in men with lower urinary tract symptoms: a pilot study, *Urol Int* 2006;76:304-8.

18 Nickel JC, Barkin J, Koch C, et al. Finasteride monotherapy maintains stable lower urinary tract symptoms in men with benign prostatic hyperplasia following cessation of alpha blockers, *Can Urol Assoc J* 2008;2:16-21.

19 Yokoyama T, Watanabe T, Saika T, et al. Natural course of lower urinary tract symptoms following discontinuation of alpha-1-adrenergic blockers in patients with benign prostatic hyperplasia, *Int J Urol* 2007;14:598-601.

20 Barkin J, Guimaraes M, Jacobi G, et al. Alpha-blocker therapy can be withdrawn in the majority of men following initial combination therapy with the dual 5alpha-reductase inhibitor dutasteride, *Eur Urol* 2003;44:461-6.

21 Matsukawa Y, Takai S, Funahashi Y, et al. Effects of Withdrawing alpha1-Blocker from Combination Therapy with alpha1-Blocker and 5alpha-Reductase Inhibitor in Patients with Lower Urinary Tract Symptoms Suggestive of Benign Prostatic Hyperplasia: A Prospective and Comparative Trial Using Urodynamics, *J Urol* 2017;198:905-12.

22 Lin VC, Liao CH, Kuo HC. Progression of lower urinary tract symptoms after discontinuation of 1 medication from 2-year combined alpha-blocker and 5-alpha-reductase inhibitor therapy for benign prostatic hyperplasia in men--a randomized multicenter study, *Urology* 2014;83:416-21.

23 Lee JY, Kang DH, Park SY, et al. Effect of Discontinuation of Tamsulosin in Korean Men with Benign Prostatic Hyperplasia Taking Tamsulosin and Dutasteride: An Open-Label, Prospective, Randomized Pilot Study, *Low Urin Tract Symptoms* 2012;4:35-40.

24 Liaw Y-, Kuo H-. Discontinuation of an alpha-1 blocker or 5-alpha-reductase inhibitor after combination medical treatment in patients with benign prostatic hyperplasia, *Tzu Chi Med J* 2006;18:91-6.

25 Baldwin KC, Ginsberg PC, Roehrborn CG, et al. Discontinuation of alpha-blockade after initial treatment with finasteride and doxazosin in men with lower urinary tract symptoms and clinical evidence of benign prostatic hyperplasia, *Urology* 2001;58:203-9.

26 Kuo HC. Comparative study of therapeutic effect of dibenyline, finasteride, and combination drugs for symptomatic benign prostatic hyperplasia, *Urol Int* 1998;60:85-91.

27 Barry MJ, Williford WO, Chang Y, et al. Benign prostatic hyperplasia specific health status measures in clinical research: how much change in the American Urological Association symptom index and the benign prostatic hyperplasia impact index is perceptible to patients? *J Urol* 1995;154:1770-4.

28 Park T, Choi JY. Efficacy and safety of dutasteride for the treatment of symptomatic benign prostatic hyperplasia (BPH): a systematic review and meta-analysis, *World J Urol* 2014;32:1093-105.

For peer review only

For peer review only - http://bmjopen.bmj.com/site/about/guidelines.xhtml

29 Kirby RS, Roehrborn C, Boyle P, et al. Efficacy and tolerability of doxazosin and finasteride, alone or in combination, in treatment of symptomatic benign prostatic hyperplasia: the Prospective European Doxazosin and Combination Therapy (PREDICT) trial, *Urology* 2003;61:119-26.

30 Roehrborn CG. Reporting of acute urinary retention in BPH treatment trials: importance of patient follow-up after discontinuation and case definitions, *Urology* 2002;59:811-5.

31 Jackson D, Turner R. Power analysis for random-effects meta-analysis, *Res Synth Methods* 2017;8:290-302.

32 Edelman M, Blanker M, Jellema P, et al. Patient attitudes towards deprescribing of alpha-blockers and willingness to participate in a discontinuation trial, *Neurourol Urodyn* 2017;36:S242-4.

For peer review only

Figure Captions

Figure 1. Forest plots of the IPSS when discontinuing or continuing alpha-blockers

A. Forest plot of the IPSS after 3 months for alpha-blocker discontinuation or continuation. B. Forest plot of the IPSS after 6 months for alpha-blocker discontinuation or continuation. Abbreviations: IPSS, International Prostate Symptom Score.

Figure 2. Forest plots of the Q-max when discontinuing or continuing alpha-blockers

A. Forest plot of the Q-max 3 months after alpha-blocker discontinuation or continuation. B. Forest plot of the Q-max 6 months after alpha-blocker discontinuation or continuation. Abbreviations: Q-max, peak urine flow rate.

Figure 3. Forest plot of the PVR 3 months after discontinuing or continuing alpha-blockers

Abbreviations: PVR, Post-void residual volume.

Figure 4. Forest plot of the QoL score 6 months after discontinuing or continuing alpha-blockers

Abbreviations: QoL, quality of life.

Figure 5. Forest plot of AEs after discontinuing or continuing alpha-blockers

Abbreviations: AEs, adverse events.

For peer review only - http://bmjopen.bmj.com/site/about/guidelines.xhtml

1A. IPSS - 3 months

Study or Subgroup	Discontinuation Mean	Discontinuation SD	Discontinuation Total	Continuation Mean	Continuation SD	Continuation Total	Weight	Mean Difference IV, Random, 95% CI
**Monotherapy**								
Kaplan et al	11.4	4.8	26	7.1	2.9	27	34.5%	4.30 [2.16, 6.44]
Yanardag et al	11.3	3.4	26	7.2	2.4	31	65.5%	4.10 [2.54, 5.66]
**Subtotal (95% CI)**			**52**			**58**	**100.0%**	**4.17 [2.91, 5.43]**
Heterogeneity: Tau² = 0.00; Chi² = 0.02, df = 1 (P = 0.88); I² = 0%								
Test for overall effect: Z = 6.49 (P < 0.00001)								
**Combination therapy**								
Barkin et al	11.1	6.2	137	10.3	6.2	140	78.5%	0.80 [-0.66, 2.26]
Liaw & Kuo	6.8	5	27	5.2	4.7	20	21.5%	1.60 [-1.19, 4.39]
**Subtotal (95% CI)**			**164**			**160**	**100.0%**	**0.97 [-0.32, 2.27]**
Heterogeneity: Tau² = 0.00; Chi² = 0.25, df = 1 (P = 0.62); I² = 0%								
Test for overall effect: Z = 1.47 (P = 0.14)								

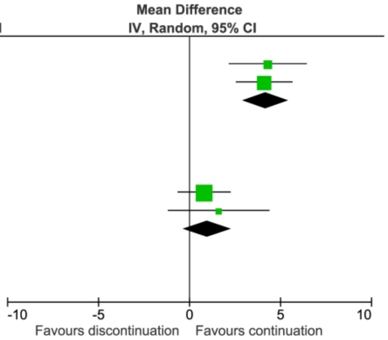

1B. IPSS - 6 months

Study or Subgroup	Discontinuation Mean	Discontinuation SD	Discontinuation Total	Continuation Mean	Continuation SD	Continuation Total	Weight	Mean Difference IV, Random, 95% CI
**Combination therapy**								
Lee et al	11.1	5.4	33	10.9	5.6	36	67.1%	0.20 [-2.40, 2.80]
Liaw & Kuo	7.9	6.7	27	6.6	6.2	20	32.9%	1.30 [-2.41, 5.01]
**Subtotal (95% CI)**			**60**			**56**	**100.0%**	**0.56 [-1.57, 2.69]**
Heterogeneity: Tau² = 0.00; Chi² = 0.23, df = 1 (P = 0.63); I² = 0%								
Test for overall effect: Z = 0.52 (P = 0.60)								

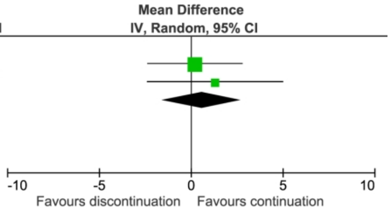

Figure 1. Forest plots of the IPSS when discontinuing or continuing alpha-blockers

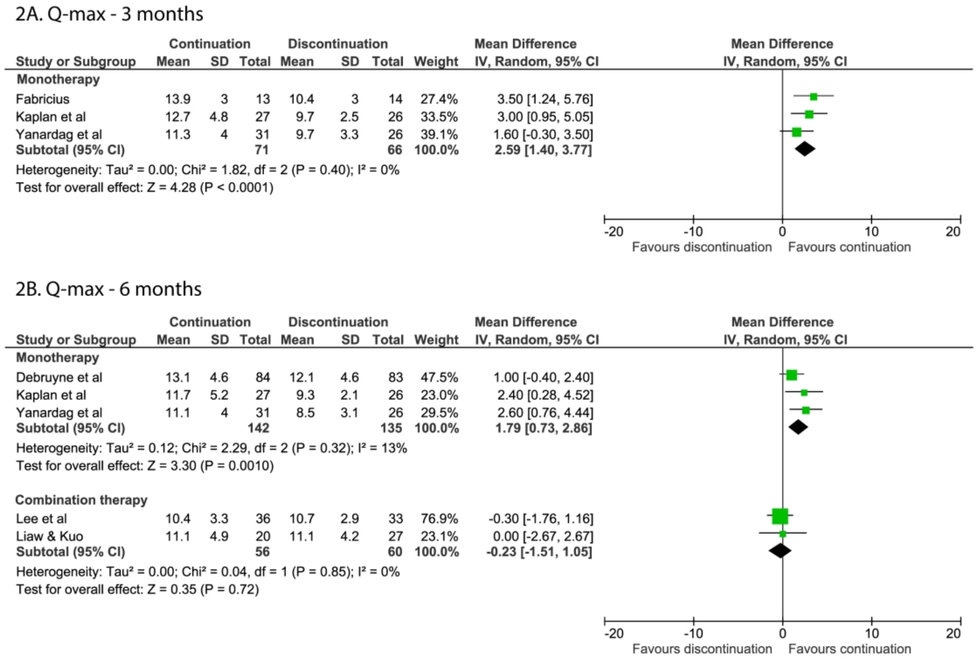

Figure 2. Forest plots of the Q-max when discontinuing or continuing alpha-blockers

PVR - 3 months

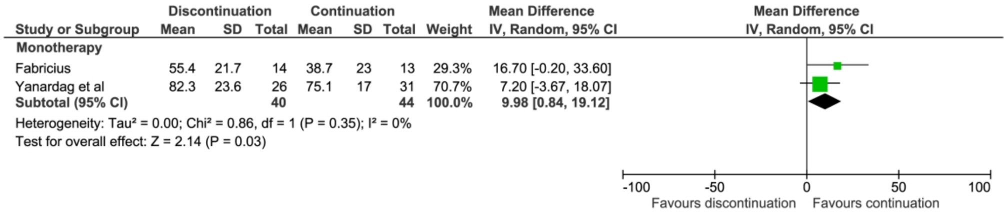

Figure 3. Forest plot of the PVR 3 months after discontinuing or continuing alpha-blockers

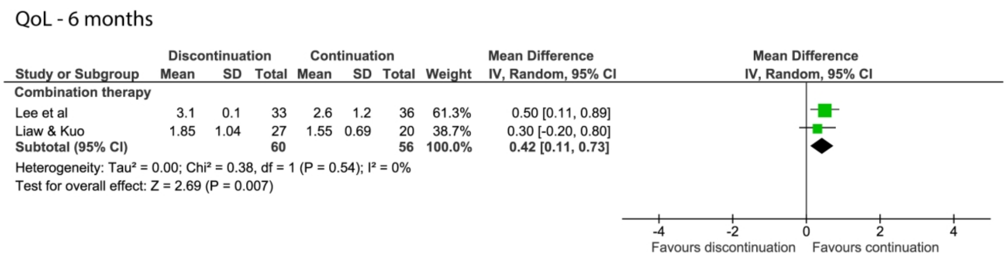

Figure 4. Forest plot of the QoL score 6 months after discontinuing or continuing alpha-blockers

Adverse events

Study or Subgroup	Discontinuation Events	Discontinuation Total	Continuation Events	Continuation Total	Weight	Risk Difference M-H, Random, 95% CI
**Monotherapy**						
Debruyne et al	39	83	40	84	5.0%	-0.01 [-0.16, 0.15]
Fabricius	0	14	0	13	6.5%	0.00 [-0.13, 0.13]
Kaplan et al	2	26	2	27	5.7%	0.00 [-0.14, 0.15]
Yanardag et al	2	26	3	31	5.4%	-0.02 [-0.17, 0.13]
**Subtotal (95% CI)**		**149**		**155**	**22.6%**	**-0.01 [-0.08, 0.07]**
Total events	43		45			
Heterogeneity: Tau² = 0.00; Chi² = 0.06, df = 3 (P = 1.00); I² = 0%						
Test for overall effect: Z = 0.15 (P = 0.88)						
**Combination therapy**						
Barkin et al	4	148	10	149	50.2%	-0.04 [-0.09, 0.01]
Matsukawa	2	66	3	66	27.2%	-0.02 [-0.08, 0.05]
**Subtotal (95% CI)**		**214**		**215**	**77.4%**	**-0.03 [-0.07, 0.01]**
Total events	6		13			
Heterogeneity: Tau² = 0.00; Chi² = 0.37, df = 1 (P = 0.54); I² = 0%						
Test for overall effect: Z = 1.59 (P = 0.11)						
**Total (95% CI)**		**363**		**370**	**100.0%**	**-0.03 [-0.06, 0.01]**
Total events	49		58			
Heterogeneity: Tau² = 0.00; Chi² = 0.86, df = 5 (P = 0.97); I² = 0%						
Test for overall effect: Z = 1.47 (P = 0.14)						
Test for subgroup differences: Chi² = 0.39, df = 1 (P = 0.53), I² = 0%						

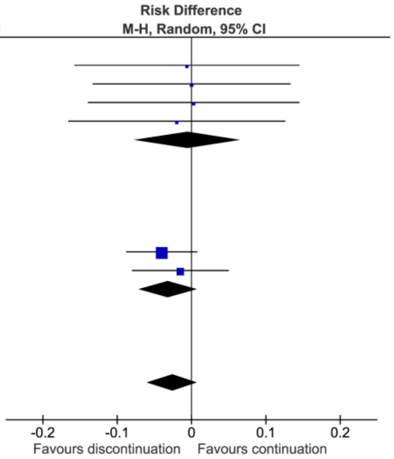

Figure 5. Forest plot of AEs after discontinuing or continuing alpha-blockers

Supplementary File 1. Search terms

Search terms

(Adrenergic alpha blockers [MeSH] OR Adrenergic alpha blockers OR alpha blockers OR doxazosin OR terazosin OR silodosin OR tamsulosin OR alfuzosin) AND (LUTS [MeSH] OR Prostatic Hyperplasia [MeSH] OR LUTS OR BPH OR lower urinary tract symptoms OR benign prostate hypertrophy OR benign prostate enlargement) AND (discontinu* OR interrup* OR cessa* OR stop* OR withdra* OR intermit*).

For peer review only

Supplementary File 2. Flow diagram of inclusion and exclusion of studies

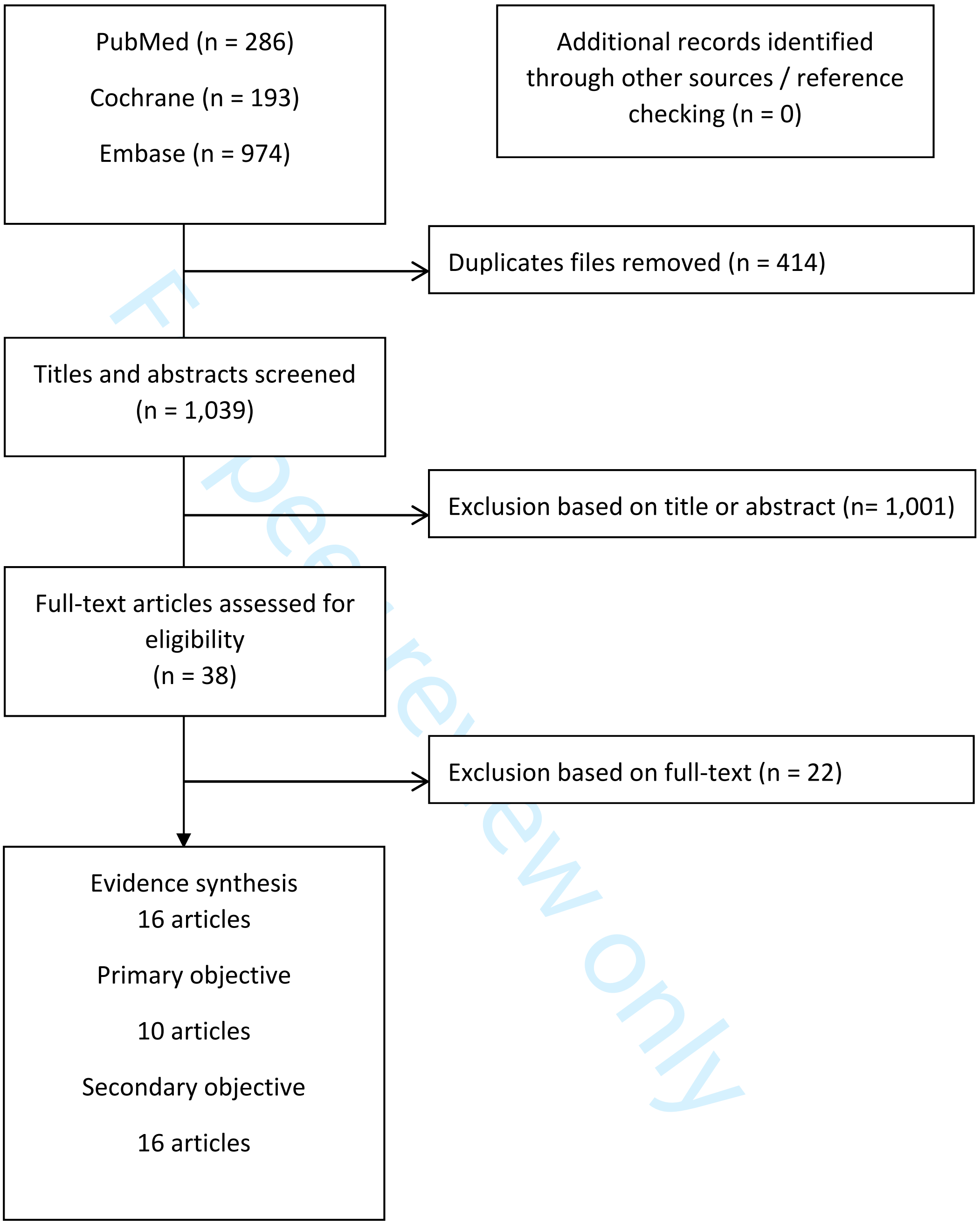

Supplementary File 3. Imputation of SD data from a previous meta-analysis

**Tool**	Ref.	Main author	Groups	N	SD	Pooled SD by study	Pooled SD overall
**IPSS**		**N = 12 studies**					
	49	Chapple	no data				
	73	Djavan	no data				
	147	Kirby	1	250	5.8	6.3	
			2	239	6.2		
			3	265	6.2		
			4	253	6.9		
	160	Lepor	no data				
	163	Lepor	no data				
	191	McConnell	no data				
	201	Mohanty	placebo	33	4	4.2	
			AB	36	4.4		
	208	Narayan	no data				
	254	Roehrborn	no data				
	255	Roehrborn	no data				
	262	Roehrborn	no data				
	305	van Kerrebroeck	N/A				
							**6.2**
**Q-max**		**N = 21 studies**					
	6	Abrams	N/A				
	33	Brawer	no data				
	50	chapple	no data				
	51	chapple	meta-analysis				
	53	christensen	N/A				
	79	elhilali	no data				
	101	gillenwater	no data				
	138	Kawabe	N/A				
	147	kirby	1	250	4.9		
			2	239	4.7	4.7	
			3	265	5.1		
			4	253	4.2		
	160	Lepor	no data				
	161	Lepor	no data				
	163	Lepor	no data				
	169	Lloyd	1	20	3.6		
			2	19	3.5	3.5	
			3	19	3.9		
			4	22	2.8		
	184	Martorana	N/A				
	201	Mohanty	placebo	33	2.6		
			AB	36	4.6	3.8	

For peer review only

	208	Narayan	no data				
	254	Roehrborn	no data				
	255	Roehrborn	no data				
	262	Roehrborn	no data				
	269	Schulman	N/A				
	305	van Kerrebroeck	N/A				
							**4.6**
**QoL**		**N = 5**					
	49	chapple	placebo	350	1		
			ocas 0.4	354	1.1	1.1	
			mr 0.4	699	1.1		
			ocas 0.8	706	1.1		
	160	Lepor	no data				
	254	Roehrborn	no data				
	255	Roehrborn	placebo	763	1.1		
			alfuzosin	759	1.1	1.1	
	305	van Kerrebroeck	N/A				
							**1.1**

In total, 12 studies assessed the IPSS, 21 assessed Q-max, and 5 assessed the IPSS QoL domain. Only studies that reported an SD at follow-up were included for the IPSS and Q-max data. No follow-up SDs were available for the QoL data, so baseline SDs were used. The SDs were imputed based on Table 6-35 from the NICE guideline on the management of LUTS [ref. 3].

Abbreviations: IPSS, International Prostate Symptom Score; N/A, article not accessible; Q-max, Peak urine flow rate; QoL, Quality of life; SD, standard deviation.

For peer review only

Supplementary File 4. Results of the risk of bias assessment

	Random sequence generation (selection bias)	Allocation concealment (selection bias)	Blinding of participants and personnel (performance bias)	Blinding of outcome assessment (detection bias)	Incomplete outcome data (attrition bias)	Selective reporting (reporting bias)	Other bias
Barkin et al	?	?	+	+	+	+	+
Debruyne et al	+	+	+	+	+	+	+
Fabricius	?	?	+	+	+	+	+
Gerber et al	−	−	−	−	−	+	+
Kaplan et al	−	−	−	−	+	+	+
Lee et al	+	+	−	−	−	+	+
Liaw & Kuo	−	?	−	−	?	+	?
Lin et al	+	?	−	−	+	+	+
Matsukawa	+	+	−	−	+	+	+
Yanardag et al	?	?	−	?	+	+	+

view only

PRISMA 2009 Checklist

Section/topic	#	Checklist item	Reported on page #
**TITLE**			
Title	1	Identify the report as a systematic review, meta-analysis, or both.	1
**ABSTRACT**			
Structured summary	2	Provide a structured summary including, as applicable: background; objectives; data sources; study eligibility criteria, participants, and interventions; study appraisal and synthesis methods; results; limitations; conclusions and implications of key findings; systematic review registration number.	2
**INTRODUCTION**			
Rationale	3	Describe the rationale for the review in the context of what is already known.	4
Objectives	4	Provide an explicit statement of questions being addressed with reference to participants, interventions, comparisons, outcomes, and study design (PICOS).	4
**METHODS**			
Protocol and registration	5	Indicate if a review protocol exists, if and where it can be accessed (e.g., Web address), and, if available, provide registration information including registration number.	4
Eligibility criteria	6	Specify study characteristics (e.g., PICOS, length of follow-up) and report characteristics (e.g., years considered, language, publication status) used as criteria for eligibility, giving rationale.	5
Information sources	7	Describe all information sources (e.g., databases with dates of coverage, contact with study authors to identify additional studies) in the search and date last searched.	5
Search	8	Present full electronic search strategy for at least one database, including any limits used, such that it could be repeated.	Supplement
Study selection	9	State the process for selecting studies (i.e., screening, eligibility, included in systematic review, and, if applicable, included in the meta-analysis).	5-6
Data collection process	10	Describe method of data extraction from reports (e.g., piloted forms, independently, in duplicate) and any processes for obtaining and confirming data from investigators.	5-6
Data items	11	List and define all variables for which data were sought (e.g., PICOS, funding sources) and any assumptions and simplifications made.	5-6
Risk of bias in individual studies	12	Describe methods used for assessing risk of bias of individual studies (including specification of whether this was done at the study or outcome level), and how this information is to be used in any data synthesis.	6
Summary measures	13	State the principal summary measures (e.g., risk ratio, difference in means).	6
Synthesis of results	14	Describe the methods of handling data and combining results of studies, if done, including measures of consistency (e.g., I^2) for each meta-analysis.	6

For peer review only

PRISMA 2009 Checklist

Section/topic	#	Checklist item	Reported on page #
Risk of bias across studies	15	Specify any assessment of risk of bias that may affect the cumulative evidence (e.g., publication bias, selective reporting within studies).	6
Additional analyses	16	Describe methods of additional analyses (e.g., sensitivity or subgroup analyses, meta-regression), if done, indicating which were pre-specified.	NA
**RESULTS**			
Study selection	17	Give numbers of studies screened, assessed for eligibility, and included in the review, with reasons for exclusions at each stage, ideally with a flow diagram.	7
Study characteristics	18	For each study, present characteristics for which data were extracted (e.g., study size, PICOS, follow-up period) and provide the citations.	7 + supplement
Risk of bias within studies	19	Present data on risk of bias of each study and, if available, any outcome level assessment (see item 12).	7+ Supplement
Results of individual studies	20	For all outcomes considered (benefits or harms), present, for each study: (a) simple summary data for each intervention group (b) effect estimates and confidence intervals, ideally with a forest plot.	Figures
Synthesis of results	21	Present results of each meta-analysis done, including confidence intervals and measures of consistency.	8-12 + supplement
Risk of bias across studies	22	Present results of any assessment of risk of bias across studies (see Item 15).	7-9+supplement
Additional analysis	23	Give results of additional analyses, if done (e.g., sensitivity or subgroup analyses, meta-regression [see Item 16]).	NA
**DISCUSSION**			
Summary of evidence	24	Summarize the main findings including the strength of evidence for each main outcome; consider their relevance to key groups (e.g., healthcare providers, users, and policy makers).	12-13
Limitations	25	Discuss limitations at study and outcome level (e.g., risk of bias), and at review-level (e.g., incomplete retrieval of identified research, reporting bias).	13-14
Conclusions	26	Provide a general interpretation of the results in the context of other evidence, and implications for future research.	14
**FUNDING**			
Funding	27	Describe sources of funding for the systematic review and other support (e.g., supply of data); role of funders for the systematic review.	3

From: Moher D, Liberati A, Tetzlaff J, Altman DG, The PRISMA Group (2009). Preferred Reporting Items for Systematic Reviews and Meta-Analyses: The PRISMA Statement. PLoS Med 6(7): e1000097.

For peer review only - http://bmjopen.bmj.com/site/about/guidelines.xhtml

For peer review only

PRISMA 2009 Checklist

doi:10.1371/journal.pmed1000097 For more information, visit: www.prisma-statement.org. Page 2 of 2

For peer review only

BMJ Open

Discontinuation of alpha-blocker therapy in men with lower urinary tract symptoms: A systematic review and meta-analysis

Journal:	*BMJ Open*
Manuscript ID	bmjopen-2019-030405.R2
Article Type:	Original research
Date Submitted by the Author:	22-Oct-2019
Complete List of Authors:	van der Worp, Henk; University of Groningen, University medical center groningen, Department of General Practice and Elderly Care Medicine<br>Jellema, Petra; University of Groningen, University medical center groningen, Department of General Practice and Elderly Care Medicine<br>Hordijk, Ilse; Isala Hospitals, urology<br>Lisman-van Leeuwen, Yvonne; University of Groningen, University medical center groningen, Department of General Practice and Elderly Care Medicine<br>Korteschiel, Lisa; Isala Hospitals, urology<br>Steffens, Martijn ; Isala Hospitals, urology<br>Blanker, Marco; University of Groningen, University medical center groningen, Department of General Practice and Elderly Care Medicine
**Primary Subject Heading**:	Urology
Secondary Subject Heading:	General practice / Family practice
Keywords:	alpha-blockers, discontinuation, lower urinary tract symptoms, meta-analysis

SCHOLARONE™
Manuscripts

Discontinuation of alpha-blocker therapy in men with lower urinary tract symptoms: A systematic review and meta-analysis

Henk van der Worp, PhD, senior-researcher (0000-0001-5545-4155) [1] , Petra Jellema, PhD, senior-researcher and epidemiologist [1], Ilse Hordijk, MD [3], Yvonne Lisman-van Leeuwen, PhD, epidemiologist [1], Lisa Korteschiel, MD [2], Martijn G Steffens, MD PhD, urologist [2], Marco H Blanker, MD PhD, GP and epidemiologist [1]

1. Department of General Practice and Elderly Medicine, University of Groningen, University Medical Centre Groningen, Groningen, the Netherlands
2. Department of Urology, Isala Clinics Zwolle, Zwolle, the Netherlands

Correspondence to:

Dr. Henk van der Worp

Department of General Practice and Elderly Medicine, University of Groningen, University Medical Centre Groningen, FA21, PO Box 196, 9700AD, Groningen, the Netherlands

h.van.der.worp@umcg.nl

+31 503615020

Word count: 3499

Keywords: alpha-blockers; discontinuation; lower urinary tract symptoms; meta-analysis

For peer review only

For peer review only - http://bmjopen.bmj.com/site/about/guidelines.xhtml

ABSTRACT

Objectives: We aimed to synthesize the available data for the effect of stopping alpha-blocker therapy among men with lower urinary tract symptoms. The focus was on symptom, uroflowmetry, and quality of life outcomes, but we also reviewed the adverse events and the number of patients who restarted therapy.

Data Sources: We searched MEDLINE/PubMed, EMBASE/Ovid, and The Cochrane Central Register of Controlled Trials from inception to May 2018

Eligibility criteria: We selected studies regardless of study design in which men were treated with an alpha-blocker for at least 3 months and in which the effects of alpha-blocker discontinuation were subsequently studied. Only controlled trials were used for the primary objective.

Data extraction and synthesis: Two reviewers independently extracted data and assessed the risk of bias for the controlled studies only, using the Cochrane Collaboration's tool for assessing risk of bias. Data were pooled using random-effects meta-analyses.

Results: We identified ten studies (1,081 participants) assessing the primary objective. Six studies (733 participants) assessed differences in adverse events between continuation and discontinuation and six studies (501 participants) reported the numbers of subjects that restarted treatment after discontinuation. No studies in primary care were identified. After discontinuing monotherapy, symptom scores increased and peak flow rates decreased at 3 and 6 months, but not at 12 months; however, neither parameter changed when alpha-blockers were stopped during combination therapy. Small differences in post-void residual volumes and quality of life scores were considered clinically irrelevant. We also found that 0%–49% of patients restarted after stopping alpha-blocker therapy and that adverse events did not increase with discontinuation.

Conclusions: Discontinuing alpha-blocker monotherapy leads to a worsening compared with

For peer review only

continuing therapy. Discontinuing the alpha-blocker after combination therapy had no significant effects on outcomes in either the short or long term.

Discontinuation may be appropriate for the frail, elderly, or those with concomitant illness or polypharmacy. However, studies in primary care are lacking.

Registration: PROSPERO database (CRD42016032648)

https://www.crd.york.ac.uk/prospero/display_record.php?RecordID=32648

For peer review only

Strengths and limitations of the study:

- This is the first systematic review that synthesizes the literature concerning alpha-blocker discontinuation.
- The review was conducted in accordance with the PRISMA-guidelines.
- The number of studies that could be included was limited and the risk of bias was high for most outcomes preventing the drawing of firm conclusions.

Role of funding sources

This research received no specific grant from any funding agency in the public, commercial or not-for-profit sectors

Competing interest declaration

All authors have completed the ICMJE uniform disclosure form at www.icmje.org/coi_disclosure.pdf and declare: no support from any organisation for the submitted work; no financial relationships with any organisations that might have an interest in the submitted work in the previous three years; no other relationships or activities that could appear to have influenced the submitted work.

INTRODUCTION

For peer review only - http://bmjopen.bmj.com/site/about/guidelines.xhtml

Alpha-blockers are the first-choice treatment for men with moderate-to-severe lower urinary tract symptoms (LUTS) because of their proven, but small, superiority over placebo,[1-3] but their use can associated with dizziness, orthostatic hypotension, and increased fall risk.[3, 4] This may be especially problematic in the elderly, who often have polypharmacy and multi-morbidity. Given the natural course of LUTS, with 30% of patients showing improvement over time,[5] it may be appropriate to consider discontinuation of alpha-blocker therapy, especially in the elderly. There are no clear data on the effect of this approach but the guideline on male LUTS for Dutch general practitioners advises that alpha-blocker therapy be discontinued after 3–6 months, followed by symptom review.[2] By contrast, guidelines followed by urologists do not advocate routine discontinuation,[1, 6] though the European Association of Urology (EAU) do mention that alpha-blocker discontinuation may be considered after 6 months in the context of combination therapy.[1]

A number of researchers have studied the effects of discontinuing alpha-blockers, but to date, there has been no synthesis of this literature.

We performed a systematic review and meta-analysis to obtain data about the effect of discontinuing alpha-blockers on male LUTS. Our primary objective was to compare the effects of discontinuing therapy with those of continuing therapy. Secondary objectives were (1) to determine the proportion of men who restart alpha-blocker therapy and (2) to determine the possible adverse effects of both discontinuation and continuation.

METHODS

We completed this review according to the Preferred Reporting Items for Systematic Reviews and Meta-Analyses (PRISMA) and registered the protocol in the PROSPERO database (CRD42016032648) http://www.crd.york.ac.uk/PROSPERO/display_record.php?ID=CRD42016032648.

Selection criteria

For peer review only - http://bmjopen.bmj.com/site/about/guidelines.xhtml

We selected studies in which men were treated with an alpha-blocker for at least 3 months and in which the effects of alpha-blocker discontinuation were subsequently studied. For the primary objective, only randomized controlled trials (RCTs, including quasi-randomized trials) and non-randomized trials (NRTs) that compared alpha-blocker discontinuation to continuation were selected. For the secondary objectives, we also included uncontrolled studies. At all stages, we excluded studies written in languages other than Dutch, English, French, or German.

Outcome measures

The following outcomes were used for the primary objective: symptom scores, such as the International Prostate Symptom Score (IPSS); urinary flow rates; post-void residual urine volume (PVR); and quality of life (QoL). For the secondary objectives, we calculated the percentage of patients who restarted alpha-blocker therapy and the numbers of adverse events (AEs) in the continuation and discontinuation groups.

Search methods for identification of studies

We searched MEDLINE/PubMed, EMBASE/Ovid, and The Cochrane Central Register of Controlled Trials using search terms covering LUTS, alpha-blockers, and discontinuation (see Supplementary File 1 for detailed information). We ran the searches in January 2016 and updated them in July 2017 and May 2018. The reference lists of relevant articles were also screened to identify additional eligible studies. All duplicate files were removed before the titles and abstracts of the remaining records were independently screened by three reviewers [IH, LK, MB] and classified as "inclusion," "exclusion," or "uncertain." Next, the same reviewers independently applied the selection criteria to the full-text papers of all records classified into the inclusion or uncertain groups, and decided whether to include or exclude the research. Discrepancies in the selection procedure were resolved by consensus.

Data extraction process

Two authors [HW, IH] independently performed data extraction using standardized forms. We extracted the following data: 1) the participant characteristics; 2) the interventions used; 3) the

For peer review only - http://bmjopen.bmj.com/site/about/guidelines.xhtml

For peer review only

primary and secondary outcomes, as well as the timing of the outcome assessment; and 4) the study design. If possible, we extracted data by allocated intervention to allow an intention-to-treat analysis. Discrepancies were resolved by re-examination and discussion of the full-text papers or by consultation with a third author [MB].

Risk of bias

Two reviewers [HW, YL] independently assessed the risk of bias using the Cochrane Collaboration's tool for assessing risk of bias.[7] This tool includes six domains, as follows: selection bias, performance bias, detection bias, attrition bias, reporting bias, and other bias. Only RCTs and NRTs were assessed, because these were used for the primary objective. Discrepancies in the risk of bias assessment were resolved by consensus or arbitration with a third party [MB]. Risk of bias was described for the five domains (selection, performance, attrition, reporting, and other) and summarized across studies and outcomes.[7]To ascertain graphically the existence of publication bias, the construction of funnel plots was planned in case at least 10 studies were included.

Data analysis

Data were analyzed using Review Manager, Version 5.3.[8] We calculated the risk difference and 95% confidence intervals (CIs) for dichotomous variables (inverse-variance method) and the mean differences (MD) with 95% CIs for continuous variables (Mantel-Haenszel method). For AEs, we also calculated the rate of AEs per 1,000 patient-days, based on the sample sizes and follow-up times.

Clinical heterogeneity was assessed by checking the characteristics of participants and interventions. Statistical heterogeneity was assessed by visual inspection of the forest plots and of the results of statistical testing for heterogeneity (I^2 statistic). We pooled data if we identified two or more studies with an I^2 of <40%,[9] using a random effects model. Data from both RCTs and NRTs were pooled. Synthesizing and pooling were done separately for monotherapy and combination therapy. If the data for pooling were only presented in figures (e.g., standard deviations), it was extracted from those figures. If data was not present at all in the article, we contacted the authors if

For peer review only - http://bmjopen.bmj.com/site/about/guidelines.xhtml

the article had been published in the past 10 years. If data could not be obtained in this way, we imputed data from a previous meta-analysis on the efficacy of alpha-blockers,[3] as described in the Cochrane Handbook.[9, 10]

The following cut-off values were used to define the minimal clinical important difference (MCID): 2.7 points for IPSS,[11] 2mL/s for Q-max,[3] and 0.5 points for the IPSS-QoL scores.[3] The MCID is the smallest change in a treatment outcome that an individual patient would identify as important and which would indicate a change in the patient's management.

Patient and public involvement

This study was performed without patient involvement. Patients were not invited to comment on the study design and were not consulted to interpret the results. Patients were not invited to contribute to the writing or editing of this document for readability or accuracy.

RESULTS

The searches yielded 1,039 publications (Supplementary File 2), of which 16 with a total of 1,823 participants were included (Table 1). All included studies were performed in secondary or tertiary care. Nine studies (772 participants) reported discontinuing alpha-blocker monotherapy: two double-blind RCTs,[12, 13] two open-label RCTs,[14, 15] one NRT,[16] and four uncontrolled studies.[17-20] Six studies (980 participants) reported discontinuing alpha-blockers used in combination therapy: one double-blind RCT,[21] four open-label RCTs,[22-25] and one uncontrolled study.[26] Finally, one uncontrolled study (N = 71) reported discontinuing both alpha-blocker monotherapy and combination therapy.[27]

Two of the included studies randomized patients into three groups: a discontinuation group, a continuation group, and a third group that continued with alternate-day use of alpha-blockers.[14, 15] We only used the data from the discontinuation and continuation groups.

For peer review only - http://bmjopen.bmj.com/site/about/guidelines.xhtml

Table 1. Characteristics of the controlled and uncontrolled studies of monotherapy and combination therapy

Authors	Design	Type of AB	Daily dose	AB Use	No. Patients	Age	IPSS at Baseline	Measured Outcomes		Follow-Up
				(Before Stopping)	(Stop Phase)	(Mean)	(Mean)	Primary	Secondary	(Months)
**Controlled trials**										
Monotherapy										
Fabricius et al. 1990 [13]	Double-blind RCT	TER	10 mg	24 wk	27	68	-	Q-max, Q-avg, PVR,	AEs	3
Debruyne et al. 1996 [12]	Double-blind RCT	TER	5 mg/10 mg	26 wk	167	63.6	19.1	IPSS, Q-max, QoL	AEs	6
Gerber et al. 1997 [16]	NRT	DOX	4 mg	3 mo	37	65	C=20.9; DC=21.5	IPSS	Restart	12
Kaplan et al. 1998 [15]	Open-label RCT (quasi-randomized)	ALF	7.5 mg	3 mo	53	60.5	15.6	IPSS, Q-max	AEs	3/6
Yanardag et al. 2005 [14]	Open-label RCT	TAM	0.4 mg	3 mo	57	61.3	12.3	IPSS, Q-max, Q-avg, PVR	AEs	3/6
Combination therapy										
Barkin et al. 2003 [21]	Double-blind RCT	TAM	0.4 mg	24 wk	277	C=67.6; DC=66.9	C=16.4; DC=16.5	IPSS, QoL	AEs	3
Liaw & Kuo 2006 [25]	Open-label RCT (quasi-randomized)	TAM	0.2 – 0.4 mg	1 yr	47	C=70.7; DC=72.1	15.6	IPSS, Q-max, QoL	Restart	3/6/12
Lee et al. 2012 [24]	Open-label RCT	TAM	0.2 mg	48 wk	69	68	15.3	IPSS, Q-max, QoL	Restart, AEs	6
Lin et al. 2014 [23]	Open-label RCT	DOX	4 mg	2 yrs	230	75	C=13.1; DC=15.6	IPSS, Q-max, PVR	Restart	12
Matsukawa et al. 2017 [22]	Open-label RCT	SIL	8 mg	12 mo	117	C=70.1; DC=69.1	C=17.4; DC=17.2	IPSS, Q-max, PVR, QoL	AEs	12
**Uncontrolled studies**										
Monotherapy										
Kobayashi et al. 2006 [18]	CS	TAM	0.2 mg	28.5 ± 26.8 mo	33	70.4	16.3		Restart	6
Yokoyama et al. 2007 [20]	CS	NAF/ TAM /URA	25-50 mg/0.2 mg/30 mg	2–200 mo	60	70 (median)	15.9		Restart	12
Nickel et al. 2008 [19]	CS	ALF/DOX/ TAM	No data	9 mo	220	66.1 (total sample)	19.9		Restart	9

For peer review only - http://bmjopen.bmj.com/site/about/guidelines.xhtml

		/TER							
Chung et al. 2013 [17]	CS	ALF	10 mg	12 wk	58	68.6 (total sample)	16.7 (total sample)	Restart	6
Combination therapy									
Baldwin et al. 2001 [26]	CS	DOX	2-8 mg	3–12 mo	240	66 (total sample)	Range: 20–33 (total study sample)	Restart	1
*Both**									
Kuo 1998 [27]	CS	DIB	20 mg	6 mo	ABM=71; ABC=65	ABM=66.3; ABC=66.8	ABM=21.2; ABC=22.5	Restart	1

AB, alpha-blocker; ABC, alpha-blocker combination treatment; ABM, alpha-blocker monotherapy; AEs, adverse events; ALF = Alfuzosin; C, continuation group; CS, cohort study; DC, discontinuation group; DIB = Dibenyline; DOX = Doxazosin; NAF = Naftopidil; NRT, non-randomized controlled trial; ns, not stated; PVR, post voided residual urine volume; Q-avg, average urine flow rate; Q-max, peak urine flow rate; SIL = Silodosin; TAM = Tamsulosin; TER = Terazosin; URA = Urapidil.

* both monotherapy and combination therapy discontinuation

For peer review only

In another three studies, the data required for pooling were missing.[12, 13, 21] Because these studies had been published over 15 years previously, no efforts were made to contact the authors. For one of the studies, means and standard deviations could be obtained from the figures.[13] For the other two studies,[12, 21] the standard deviations were missing and were imputed from the results of a previous meta-analysis (see Supplementary File 3).[3] No studies were excluded form pooling based on statistical heterogeneity.

Risk of bias

Most of the included studies had risks of bias (Supplementary File 4). The most common were lack of blinding and randomization, with only three out of ten studies having a “low risk” for these items. There was no evidence of reporting bias in any of the included studies. The summary of bias by study indicated that only one study had low risk of bias,[12] while two studies had unclear risks of bias,[13, 21] and the remaining studies had high risks of bias.[14-16, 22-25] As a result, risk of bias was high for all but one outcome.

We did not construct funnel plots to ascertain the existence of publication bias graphically, as the number of included studies in each meta-analysis was less than 10.

Effects of alpha-blocker discontinuation

Five studies of monotherapy (n = 341) [12, 13, 15, 16] and five studies of combination therapy (n=740) [21-25] were used for the primary research objective. Only three provided data on the number of patients who did not comply with the intervention and who restarted alpha-blocker use after discontinuation. Two of these provided a per protocol analysis excluding those patients [24, 25] and the third provided an intention-to-treat analysis for categorized variables, with a per protocol analysis for the raw outcomes,[23] effectively precluding an intention-to-treat analysis. Because all included studies reported outcomes at 3, 6, or 12 months, we compared outcomes at these time points.

Symptom scores

For peer review only - http://bmjopen.bmj.com/site/about/guidelines.xhtml

For peer review only

All but one study [13] assessed symptoms with the IPSS questionnaire, or its predecessor the AUA symptom score (n = 1,054).

By 3 months after discontinuing alpha-blocker monotherapy, symptoms increased in the discontinuation group compared with the continuation group (MD = 4.17; 95% CI 2.91 to 5.43),[14, 15] whereas there was no difference between the continuation and discontinuation groups in the studies of combination therapy (MD = 0.97; 95% CI -0.32 to 2.27, Figure 1A).[21, 25]

After 6 months, two RCTs and one NRT on monotherapy found a significant worsening of symptoms (differences varying from 2.0 to 5.8 points) in subjects that discontinued alpha-blockers.[12, 14, 15] No difference was found for studies on combination therapy after 6 months (MD = 0.56; 95% CI -1.57;2.69, Figure 1B).[24, 25]

After 12 months, the one study that looked at discontinuing monotherapy found a non-significant difference of 1.2 points between groups.[16] No differences were found in two open-label RCTs that looked at alpha-blocker discontinuation after combination therapy.[22, 23] Another NRT presented data for both groups, but did not make a direct statistical comparison between groups.[25] Data could not be pooled (I^2=61%).

Peak urine flow rate

Nine studies (804 patients) assessed peak urine flow rate (Q-max): five studies for monotherapy,[12-16] and four studies for combination therapy.[22-25]

After 3 months, Q-max reduced by 2.59 mL/s (95% CI 1.40 to 3.77, Figure 2A) in those who discontinued alpha-blocker monotherapy compared with those who continued therapy.[13-15] A single study on discontinuing combination therapy found that there was an increase of 1.4 mL/s in those who discontinued compared to those who continued therapy, but the researchers did not perform a statistical comparison.[25]

After 6 months, a reduction was again found in the Q-max after discontinuing monotherapy (MD = 1.79; 95% CI 0.73 to 2.86),[12, 14, 15] but no difference was found after discontinuing in the context of combination therapy (MD -0.23; 95% CI -1.51 to 1.05, Figure 2B).[24, 25]

For peer review only - http://bmjopen.bmj.com/site/about/guidelines.xhtml

After 12 months, no differences were found in an NRT reporting on the effects of discontinuing monotherapy.[16] Three studies assessed Q-max 12 months after combination therapy. Among these, two open-label RCTs found no difference between groups:[22, 23] one study found a difference of 0.1 mL/s in favor of the continuation group and the other found that 7% fewer patients in the discontinuation group had a reduction in Q-max of >2 mL/s compared with the continuation group. Again, the NRT on combination therapy showed an increase of 2.5 mL/s after alpha-blocker discontinuation, which was not seen in the group that continued alpha-blockers, but differences were not tested.[25] Data could not be pooled (I^2 = 68%).

Average urine flow rate (Q-avg)

Data from two RCTs on monotherapy (84 patients) could not be pooled (I^2 = 70%).[13, 14] After 3 months, one RCT reported a reduction of 2.2 mL/s in subjects who discontinued therapy compared with those who continued therapy,[13] whereas no statistical testing of the difference of 0.6 mL/s between groups was performed in the other RCT.[14]

After 6 months, this second study found a difference of 0.9 mL/s between groups in favor of continuing monotherapy.[14]

Post voided residual urine volume (PVR)

PVR volume was measured in five studies (n = 468), with three measuring it after discontinuing monotherapy [13, 14, 16] and two measuring it after discontinuing the alpha-blocker in combination therapy.[22, 23]

After 3 months, discontinuing monotherapy resulted in a PVR volume increase of 9.98 mL (95% CI 0.84 to 19.12, Figure 3).[13, 14]

After 6 months, an open-label trial on discontinuing monotherapy (57 participants) also found a statistically significant difference (14.1 mL) in favor of continuing therapy.[14]

At 12 months after discontinuing monotherapy, an NRT did not find a significant difference between groups.[16] Two open-label RCTs on discontinuing combination therapy did report non-

For peer review only - http://bmjopen.bmj.com/site/about/guidelines.xhtml

significant differences: one showed a 2 mL difference between groups,[22] and the other showed that 8% more patients in the discontinuation group reported a PVR increase of >50%.[23]

Quality of life

All five studies (one of monotherapy and four of combination therapy; 677 patients) that assessed QoL used the IPSS QoL sub-score.

After 3 months, one study of combination therapy found no difference between groups (0 points).[21] In another NRT of combination therapy, a difference of 0.4 points was reported in favor of the group that discontinued therapy, but this was not statistically tested.[25]

After 6 months, one study on monotherapy found a statistically significant difference of 0.2 points in favor of those who continued alpha-blocker therapy.[12] This was the only outcome with a low risk of bias. A difference was found for the pooled studies of combination therapy (MD = 0.42; 95% CI 0.11–0.73, Figure 4).[24, 25]

After 12 months, no differences in QoL scores (only 0.1 points in favor of discontinuation) were found in an RCT of 117 participants receiving combination therapy.[22] Another NRT of patients receiving combination therapy found a difference of 0.4 in favor of continuation, but did not compare groups statistically.[25] Data could not be pooled ($I^2 = 60\%$).

Restart of prior treatment and AEs

Patients restarting treatment

Six studies (501 patients) reported data on restarting alpha-blockers after discontinuation.[16, 17, 20, 23-25] In three of these (187 patients), 7%–49% of subjects restarted alpha-blockers after 6 months.[17, 20, 24] One of these,[20] together with another three studies (374 patients), [16, 23, 25] described that 0%–33% of subjects had restarted alpha-blockers at 12 months. The highest (49%) and lowest percentages (0%) restarting therapy were found in studies of monotherapy.[16, 17] However, four of the included studies explicitly advised subjects to restart alpha-blocker use if their PVR was >100 mL,[17, 20] or if symptoms worsened.[23, 25] These studies

For peer review only - http://bmjopen.bmj.com/site/about/guidelines.xhtml

reported the highest restart rates.

Three other studies provided indirect information about restarting alpha-blocker use.[18, 26, 27] Two of these reported on successful discontinuation, defined as no increase in symptoms and no request for continuation of treatment. After 6 months, one indicated success among 69% of those receiving monotherapy.[18] Another study reported success rates of 13%–87% one month after discontinuing combination therapy, with percentages increasing as the duration of alpha-blocker use increased (ranging from 3 – 12 months).[26] Discontinuation was successful in 13%–20% of subjects who used alpha-blockers for 3 months and in 84%–87% of subjects who used them for 12 months. A third study stated that most patients whose symptoms worsened after discontinuation wished to restart their medication rather than undergo surgery.[27]

Adverse events

Nine studies provided no data on AEs during discontinuation, or if they did, provided data without a clear indication of the treatment group.[24] Another study only reported AEs during follow-up for those who discontinued alpha-blockers.[19] The six remaining studies reported 49 AEs in 363 patients who discontinued alpha-blockers and 58 AEs in 370 patients who continued to use alpha-blockers.[12-15, 21, 22] The AE rates in patients who discontinued or continued alpha-blockers were 0.13 and 0.15 per 1,000 patient-days, respectively. The pooled data showed no risk difference for AEs when discontinuing or continuing either monotherapy (risk difference = -0.01; 95% CI -0.08 to 0.07) or combination therapy (risk difference = -0.03; 95% CI -0.07 to 0.01, Figure 5).

Respiratory tract infection and urinary retention were the two most common AEs after discontinuing alpha-blockers (11 studies in total), being reported in 1%–4% of patients [12, 21] and in 1%–3% of patients,[12, 22] respectively. The incidence of these AEs did not differ between groups.

DISCUSSION

The results of this systematic review indicate that discontinuing alpha-blocker monotherapy leads to a worsening of clinical symptoms and a decrease of urinary flow rates in the short-term (3–6

For peer review only - http://bmjopen.bmj.com/site/about/guidelines.xhtml

months) compared with continuing therapy. However, after one year, no differences were found in these or other outcomes. Discontinuing the alpha-blocker after combination therapy had no significant effects on outcomes in either the short or long term.

The worsening of symptoms over the short-term after stopping monotherapy was probably relevant to clinical practice. The reported differences in the IPSS between groups exceeded the minimal clinically important difference (MCID) of 2.7 points.[11] The difference in Q-max was also clinically relevant, exceeding the MCID of 2 mL/s after 3 months (between-group difference, 2.59 mL/s),[3] but not after 6 months (1.79 mL/s). One might argue about the relevance of this outcome for patient, as men will not be able to notice a difference in flow rate at these values. The difference of 0.42 points in the QoL scores at 6 months after discontinuing combination therapy remained below the MCID of 0.5 points.[3] Although no MCID was available for PVR, we do not think that the reported mean difference of 10 mL after 3 months was clinically relevant.

The worsening of symptoms noted by 3–6 months after discontinuing monotherapy was larger than the reported improvement of symptoms after initiating therapy, which was reported to be 2.55 points (95% CI, 1.92–3.17) based on 12 RCTs with a total of 9,335 participants.[3] The magnitude of change in the present review may have been influenced by the lack of blinding of both patients and assessors in many of the studies, which will have favored the continuation groups. Men in these studies who had no clear symptom improvements are likely to have dropped out before the discontinuation phase, so the participants subsequently included in the discontinuation trials will generally have had larger treatment effects and larger changes after discontinuation. The outcomes after 12 months relied on data from a single study on discontinuing doxazosin (not a controlled-release version),[16] which has a lower efficacy than other alpha-blockers. This might explain the lack of any meaningful long-term impact.

Among patients receiving combination therapy, outcomes were not significantly different between those discontinuing and continuing alpha-blockers. Although 5-alpha-reductase inhibitors have no significant impact on LUTS severity after treatment initiation,[28, 29] their continuation

For peer review only - http://bmjopen.bmj.com/site/about/guidelines.xhtml

seems to be protective against symptom worsening after discontinuing alpha-blockers.

The results for restarting a discontinued alpha-blocker were heterogeneous, ranging widely from 0% to 49%. These conflicting findings can be explained by the differences in instructions given to patients in these studies. Indeed, participants in some studies received explicit instructions about when to restart therapy, whereas in other studies, no instructions were given. Also, subjects in cohort studies who volunteered to discontinue therapy may have had greater freedom to restart therapy than those participating in an RCT.

It was also shown that discontinuing alpha-blockers did not result in more AEs, including acute urinary retention.[30] Equally, continuation was not associated with more AEs, with neither dizziness nor orthostatic hypotension being more common.[4] This may be explained by subject drop-out due to AEs before entering the discontinuation phase. The number of patients reporting AEs in the included studies was, however, too small to draw meaningful conclusions regarding AEs.

Interpretation of our findings is hampered by some limitations. For example, the limited numbers of studies and large amount of statistical heterogeneity limited data pooling. Heterogeneity, especially on IPSS outcomes after 6 months could be explained by differences in alpha-blockers studied and baseline symptom severity differences ranging from 12 to 19 in the included studies. The limited number of RCTs also precluded sensitivity analyses, and subgroup analyses, that were planned in the original review protocol. Another limitation related to the limited number of studies is the reduction in statistical power. It has been shown that at least five studies have to be pooled to achieve a greater power than the original studies independently.[31] So, our results could also be subject to Type I error. In addition, some studies gave unclear data about treatment compliance or only presented per protocol analyses, which may have led to bias (e.g., drop-out due to severe complaints) and loss of generalizability. Another issue is that all studies were performed in secondary or tertiary care settings. This is important if we consider that in some countries, most men with LUTS are treated in primary care. The high risk of bias, which was noted for all but one outcome, also hampers the interpretation of our findings. Finally, two of the trials of

For peer review only - http://bmjopen.bmj.com/site/about/guidelines.xhtml

combination therapy compared discontinuing alpha-blockers and 5-alpha-reductase inhibitors, but the others compared discontinuing and continuing only the alpha-blocker.[23, 25]

No firm conclusions can be drawn from this review because of the low quality of the available evidence. Overall, the data suggest that there is a short-term clinical worsening of LUTS after discontinuing alpha-blocker monotherapy, as assessed by symptom scores and urinary flow rates, but that this does not increase the risk of a complicated symptom course.

Patients frequently discontinue alpha-blocker treatment in clinical practice. We have recently shown that men who continue to use alpha-blockers are typically unconcerned about stopping that therapy if advised to do so by a doctor.[32] The present review also provides evidence that the magnitude of symptom deterioration is limited, indicating that physicians can change their prescribing policy without risking harm. Indeed, the alternative approach may promote unnecessary polypharmacy, which is especially relevant in vulnerable groups. Active follow-up should then be used to monitor the need to restart alpha-blockers if symptoms worsen.

Our findings support the existing EAU guidance to consider discontinuing alpha-blockers in patients receiving combination therapy for 6 months.[1] Unfortunately, because the studies in this review were only performed in secondary care, we cannot give firm support for the recommendation of the Dutch GP guideline to review therapy after 3–6 months in primary care.[2] Symptom levels before treatment are generally lower in primary care, where conditions are typically less severe than in secondary care. Although the data from this review may be applicable to primary care, further efficacy studies and discontinuation trials are needed to assess the outcomes specific to this setting.

Acknowledgments

We thank Dr Robert Sykes (www.doctored.org.uk) for providing editorial services.

Author statement

For peer review only - http://bmjopen.bmj.com/site/about/guidelines.xhtml

For peer review only

The corresponding author attests that all listed authors meet authorship criteria and that no others meeting the criteria have been omitted.

MHB had the idea for the article. Acquisition of the data was done by IH, LK, HW and MHB. Analysis and interpretation of the data was done by HW, MHB, PJ, YL and MGS. HW and MHB wrote the manuscript. All authors critically reviewed the manuscript.

Data sharing statement

Data collected for this study will be available from the corresponding author upon request.

For peer review only

For peer review only - http://bmjopen.bmj.com/site/about/guidelines.xhtml

References

1 Gratzke C, Bachmann A, Descazeaud A, et al. EAU Guidelines on the Assessment of Non-neurogenic Male Lower Urinary Tract Symptoms including Benign Prostatic Obstruction, *Eur Urol* 2015;67:1099-109.

2 Blanker M, Klomp M, Donk M, et al. Summary of the NHG practice guideline 'Lower urinary tract symptoms in men', *Ned Tijdschr Geneeskd* 2013;157:A6178.

3 National Clinical Guideline Centre (NICE).
The management of lower urinary tract symptoms in men: NICE guideline. 2010;CG97(01/09).

4 Bird ST, Delaney JA, Brophy JM, et al. Tamsulosin treatment for benign prostatic hyperplasia and risk of severe hypotension in men aged 40-85 years in the United States: risk window analyses using between and within patient methodology, *BMJ* 2013;347:f6320.

5 Kok ET, Bohnen AM, Groeneveld FP, et al. Changes in disease specific and generic quality of life related to changes in lower urinary tract symptoms: the Krimpen study, *J Urol* 2005;174:1055-8.

6 McVary KT, Roehrborn CG, Avins AL, et al. Update on AUA guideline on the management of benign prostatic hyperplasia, *J Urol* 2011;185:1793-803.

7 Higgins JP, Altman DG, Gotzsche PC, et al. The Cochrane Collaboration's tool for assessing risk of bias in randomised trials, *BMJ* 2011;343:d5928.

8 Review Manager (RevMan) [Computer program]. Version 5.3. Copenhagen: The Nordic Cochrane Centre, The Cochrane Collaboration, 2014.

9 Higgins JPT, Green S (editors). Cochrane Handbook for Systematic Reviews of Interventions Version 5.1.0 [updated March 2011]. The Cochrane Collaboration 2011. Available from www.handbook.cochrane.org.

10 Furukawa TA, Barbui C, Cipriani A, et al. Imputing missing standard deviations in meta-analyses can provide accurate results, *J Clin Epidemiol* 2006;59:7-10.

11 Barry MJ, Williford WO, Chang Y, et al. Benign prostatic hyperplasia specific health status measures in clinical research: how much change in the American Urological Association symptom index and the benign prostatic hyperplasia impact index is perceptible to patients? *J Urol* 1995;154:1770-4.

12 Debruyne FM, Witjes WP, Fitzpatrick J, et al. The international terazosin trial: a multicentre study of the long-term efficacy and safety of terazosin in the treatment of benign prostatic hyperplasia. The ITT Group, *Eur Urol* 1996;30:369-76.

13 Fabricius P G PG. Efficacy of once-a-day terazosin in benign prostatic hyperplasia: a randomized, double-blind placebo-controlled clinical trial. *The Prostate.Supplement* 1990;3:85-93.

14 Yanardag H, Goktas S, Kibar Y, et al. Intermittent tamsulosin therapy in men with lower urinary tract symptoms, *J Urol* 2005;173:155-7.

For peer review only

For peer review only - http://bmjopen.bmj.com/site/about/guidelines.xhtml

15 Kaplan SA, Reis RB, Cologna A, et al. Intermittent alpha-blocker therapy in the treatment of men with lower urinary tract symptoms, *Urology* 1998;52:12-6.

16 Gerber GS, Contreras BA, Zagaja GP, et al. Doxazosin in men with lower urinary tract symptoms: urodynamic evaluation at 15 months, *Urology* 1997;50:229-33.

17 Chung JH, Lee JY, Kang DH, et al. Evaluation of patient outcome after discontinuation of alfuzosin treatment for benign prostatic hyperplasia: a multicentre, prospective study, *Int J Clin Pract* 2013;67:870-5.

18 Kobayashi M, Tokue A, Morita T. Discontinuation of tamsulosin treatment in men with lower urinary tract symptoms: a pilot study, *Urol Int* 2006;76:304-8.

19 Nickel JC, Barkin J, Koch C, et al. Finasteride monotherapy maintains stable lower urinary tract symptoms in men with benign prostatic hyperplasia following cessation of alpha blockers, *Can Urol Assoc J* 2008;2:16-21.

20 Yokoyama T, Watanabe T, Saika T, et al. Natural course of lower urinary tract symptoms following discontinuation of alpha-1-adrenergic blockers in patients with benign prostatic hyperplasia, *Int J Urol* 2007;14:598-601.

21 Barkin J, Guimaraes M, Jacobi G, et al. Alpha-blocker therapy can be withdrawn in the majority of men following initial combination therapy with the dual 5alpha-reductase inhibitor dutasteride, *Eur Urol* 2003;44:461-6.

22 Matsukawa Y, Takai S, Funahashi Y, et al. Effects of Withdrawing alpha1-Blocker from Combination Therapy with alpha1-Blocker and 5alpha-Reductase Inhibitor in Patients with Lower Urinary Tract Symptoms Suggestive of Benign Prostatic Hyperplasia: A Prospective and Comparative Trial Using Urodynamics, *J Urol* 2017;198:905-12.

23 Lin VC, Liao CH, Kuo HC. Progression of lower urinary tract symptoms after discontinuation of 1 medication from 2-year combined alpha-blocker and 5-alpha-reductase inhibitor therapy for benign prostatic hyperplasia in men--a randomized multicenter study, *Urology* 2014;83:416-21.

24 Lee JY, Kang DH, Park SY, et al. Effect of Discontinuation of Tamsulosin in Korean Men with Benign Prostatic Hyperplasia Taking Tamsulosin and Dutasteride: An Open-Label, Prospective, Randomized Pilot Study, *Low Urin Tract Symptoms* 2012;4:35-40.

25 Liaw Y-, Kuo H-. Discontinuation of an alpha-1 blocker or 5-alpha-reductase inhibitor after combination medical treatment in patients with benign prostatic hyperplasia, *Tzu Chi Med J* 2006;18:91-6.

26 Baldwin KC, Ginsberg PC, Roehrborn CG, et al. Discontinuation of alpha-blockade after initial treatment with finasteride and doxazosin in men with lower urinary tract symptoms and clinical evidence of benign prostatic hyperplasia, *Urology* 2001;58:203-9.

27 Kuo HC. Comparative study of therapeutic effect of dibenyline, finasteride, and combination drugs for symptomatic benign prostatic hyperplasia, *Urol Int* 1998;60:85-91.

28 Park T, Choi JY. Efficacy and safety of dutasteride for the treatment of symptomatic benign prostatic hyperplasia (BPH): a systematic review and meta-analysis, *World J Urol* 2014;32:1093-105.

For peer review only - http://bmjopen.bmj.com/site/about/guidelines.xhtml

For peer review only

29 Kirby RS, Roehrborn C, Boyle P, et al. Efficacy and tolerability of doxazosin and finasteride, alone or in combination, in treatment of symptomatic benign prostatic hyperplasia: the Prospective European Doxazosin and Combination Therapy (PREDICT) trial, *Urology* 2003;61:119-26.

30 Roehrborn CG. Reporting of acute urinary retention in BPH treatment trials: importance of patient follow-up after discontinuation and case definitions, *Urology* 2002;59:811-5.

31 Jackson D, Turner R. Power analysis for random-effects meta-analysis, *Res Synth Methods* 2017;8:290-302.

32 Edelman M, Blanker M, Jellema P, et al. Patient attitudes towards deprescribing of alpha-blockers and willingness to participate in a discontinuation trial, *Neurourol Urodyn* 2017;36:S242-4.

For peer review only

For peer review only - http://bmjopen.bmj.com/site/about/guidelines.xhtml

Figure Captions

Figure 1. Forest plots of the IPSS when discontinuing or continuing alpha-blockers

A. Forest plot of the IPSS after 3 months for alpha-blocker discontinuation or continuation. B. Forest plot of the IPSS after 6 months for alpha-blocker discontinuation or continuation. Abbreviations: IPSS, International Prostate Symptom Score.

Figure 2. Forest plots of the Q-max when discontinuing or continuing alpha-blockers

A. Forest plot of the Q-max 3 months after alpha-blocker discontinuation or continuation. B. Forest plot of the Q-max 6 months after alpha-blocker discontinuation or continuation. Abbreviations: Q-max, peak urine flow rate.

Figure 3. Forest plot of the PVR 3 months after discontinuing or continuing alpha-blockers

Abbreviations: PVR, Post-void residual volume.

Figure 4. Forest plot of the QoL score 6 months after discontinuing or continuing alpha-blockers

Abbreviations: QoL, quality of life.

Figure 5. Forest plot of AEs after discontinuing or continuing alpha-blockers

Abbreviations: AEs, adverse events.

For peer review only - http://bmjopen.bmj.com/site/about/guidelines.xhtml

1A. IPSS - 3 months

Study or Subgroup	Discontinuation Mean	Discontinuation SD	Discontinuation Total	Continuation Mean	Continuation SD	Continuation Total	Weight	Mean Difference IV, Random, 95% CI
**Monotherapy**								
Kaplan et al	11.4	4.8	26	7.1	2.9	27	34.5%	4.30 [2.16, 6.44]
Yanardag et al	11.3	3.4	26	7.2	2.4	31	65.5%	4.10 [2.54, 5.66]
**Subtotal (95% CI)**			**52**			**58**	**100.0%**	**4.17 [2.91, 5.43]**
Heterogeneity: Tau² = 0.00; Chi² = 0.02, df = 1 (P = 0.88); I² = 0%								
Test for overall effect: Z = 6.49 (P < 0.00001)								
**Combination therapy**								
Barkin et al	11.1	6.2	137	10.3	6.2	140	78.5%	0.80 [-0.66, 2.26]
Liaw & Kuo	6.8	5	27	5.2	4.7	20	21.5%	1.60 [-1.19, 4.39]
**Subtotal (95% CI)**			**164**			**160**	**100.0%**	**0.97 [-0.32, 2.27]**
Heterogeneity: Tau² = 0.00; Chi² = 0.25, df = 1 (P = 0.62); I² = 0%								
Test for overall effect: Z = 1.47 (P = 0.14)								

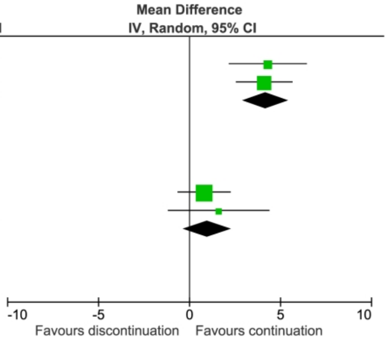

1B. IPSS - 6 months

Study or Subgroup	Discontinuation Mean	Discontinuation SD	Discontinuation Total	Continuation Mean	Continuation SD	Continuation Total	Weight	Mean Difference IV, Random, 95% CI
**Combination therapy**								
Lee et al	11.1	5.4	33	10.9	5.6	36	67.1%	0.20 [-2.40, 2.80]
Liaw & Kuo	7.9	6.7	27	6.6	6.2	20	32.9%	1.30 [-2.41, 5.01]
**Subtotal (95% CI)**			**60**			**56**	**100.0%**	**0.56 [-1.57, 2.69]**
Heterogeneity: Tau² = 0.00; Chi² = 0.23, df = 1 (P = 0.63); I² = 0%								
Test for overall effect: Z = 0.52 (P = 0.60)								

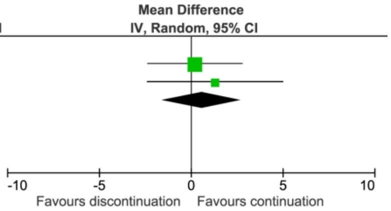

Figure 1. Forest plots of the IPSS when discontinuing or continuing alpha-blockers

2A. Q-max - 3 months

Study or Subgroup	Continuation Mean	Continuation SD	Continuation Total	Discontinuation Mean	Discontinuation SD	Discontinuation Total	Weight	Mean Difference IV, Random, 95% CI
**Monotherapy**								
Fabricius	13.9	3	13	10.4	3	14	27.4%	3.50 [1.24, 5.76]
Kaplan et al	12.7	4.8	27	9.7	2.5	26	33.5%	3.00 [0.95, 5.05]
Yanardag et al	11.3	4	31	9.7	3.3	26	39.1%	1.60 [-0.30, 3.50]
**Subtotal (95% CI)**			**71**			**66**	**100.0%**	**2.59 [1.40, 3.77]**
Heterogeneity: $Tau^2 = 0.00$; $Chi^2 = 1.82$, df = 2 (P = 0.40); $I^2 = 0\%$								
Test for overall effect: Z = 4.28 (P < 0.0001)								

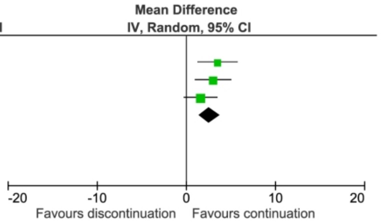

2B. Q-max - 6 months

Study or Subgroup	Continuation Mean	Continuation SD	Continuation Total	Discontinuation Mean	Discontinuation SD	Discontinuation Total	Weight	Mean Difference IV, Random, 95% CI
**Monotherapy**								
Debruyne et al	13.1	4.6	84	12.1	4.6	83	47.5%	1.00 [-0.40, 2.40]
Kaplan et al	11.7	5.2	27	9.3	2.1	26	23.0%	2.40 [0.28, 4.52]
Yanardag et al	11.1	4	31	8.5	3.1	26	29.5%	2.60 [0.76, 4.44]
**Subtotal (95% CI)**			**142**			**135**	**100.0%**	**1.79 [0.73, 2.86]**
Heterogeneity: $Tau^2 = 0.12$; $Chi^2 = 2.29$, df = 2 (P = 0.32); $I^2 = 13\%$								
Test for overall effect: Z = 3.30 (P = 0.0010)								
**Combination therapy**								
Lee et al	10.4	3.3	36	10.7	2.9	33	76.9%	-0.30 [-1.76, 1.16]
Liaw & Kuo	11.1	4.9	20	11.1	4.2	27	23.1%	0.00 [-2.67, 2.67]
**Subtotal (95% CI)**			**56**			**60**	**100.0%**	**-0.23 [-1.51, 1.05]**
Heterogeneity: $Tau^2 = 0.00$; $Chi^2 = 0.04$, df = 1 (P = 0.85); $I^2 = 0\%$								
Test for overall effect: Z = 0.35 (P = 0.72)								

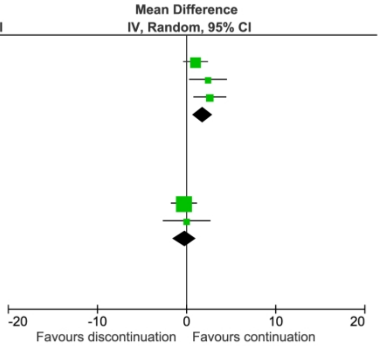

Figure 2. Forest plots of the Q-max when discontinuing or continuing alpha-blockers

PVR - 3 months

Study or Subgroup	Discontinuation Mean	Discontinuation SD	Discontinuation Total	Continuation Mean	Continuation SD	Continuation Total	Weight	Mean Difference IV, Random, 95% CI
**Monotherapy**								
Fabricius	55.4	21.7	14	38.7	23	13	29.3%	16.70 [-0.20, 33.60]
Yanardag et al	82.3	23.6	26	75.1	17	31	70.7%	7.20 [-3.67, 18.07]
**Subtotal (95% CI)**			**40**			**44**	**100.0%**	**9.98 [0.84, 19.12]**

Heterogeneity: Tau² = 0.00; Chi² = 0.86, df = 1 (P = 0.35); I² = 0%

Test for overall effect: Z = 2.14 (P = 0.03)

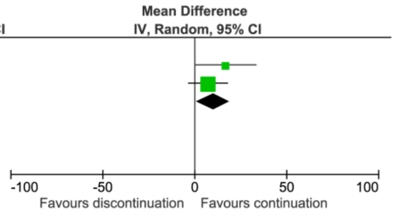

Figure 3. Forest plot of the PVR 3 months after discontinuing or continuing alpha-blockers

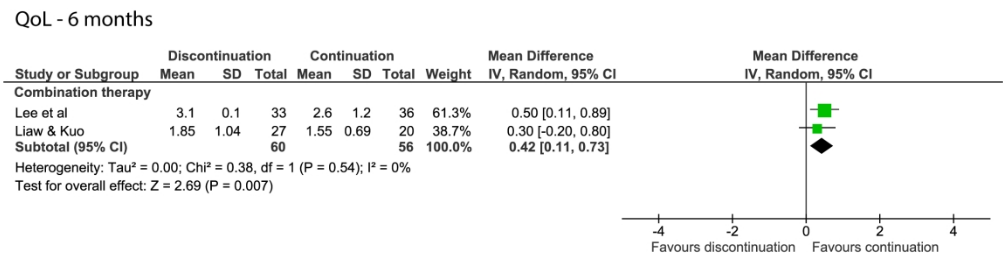

Figure 4. Forest plot of the QoL score 6 months after discontinuing or continuing alpha-blockers

Adverse events

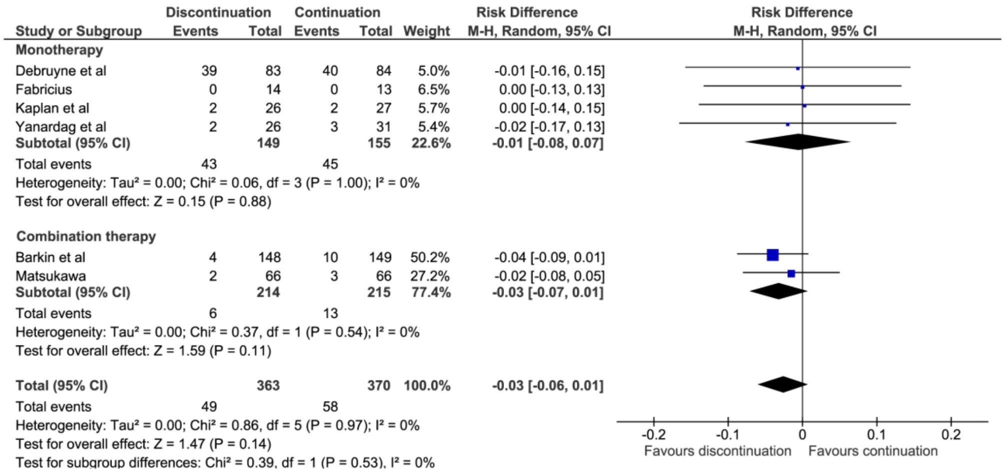

Figure 5. Forest plot of AEs after discontinuing or continuing alpha-blockers

Supplementary File 1. Search terms

Search terms

(Adrenergic alpha blockers [MeSH] OR Adrenergic alpha blockers OR alpha blockers OR doxazosin OR terazosin OR silodosin OR tamsulosin OR alfuzosin) AND (LUTS [MeSH] OR Prostatic Hyperplasia [MeSH] OR LUTS OR BPH OR lower urinary tract symptoms OR benign prostate hypertrophy OR benign prostate enlargement) AND (discontinu* OR interrup* OR cessa* OR stop* OR withdra* OR intermit*).

For peer review only

Supplementary File 2. Flow diagram of inclusion and exclusion of studies

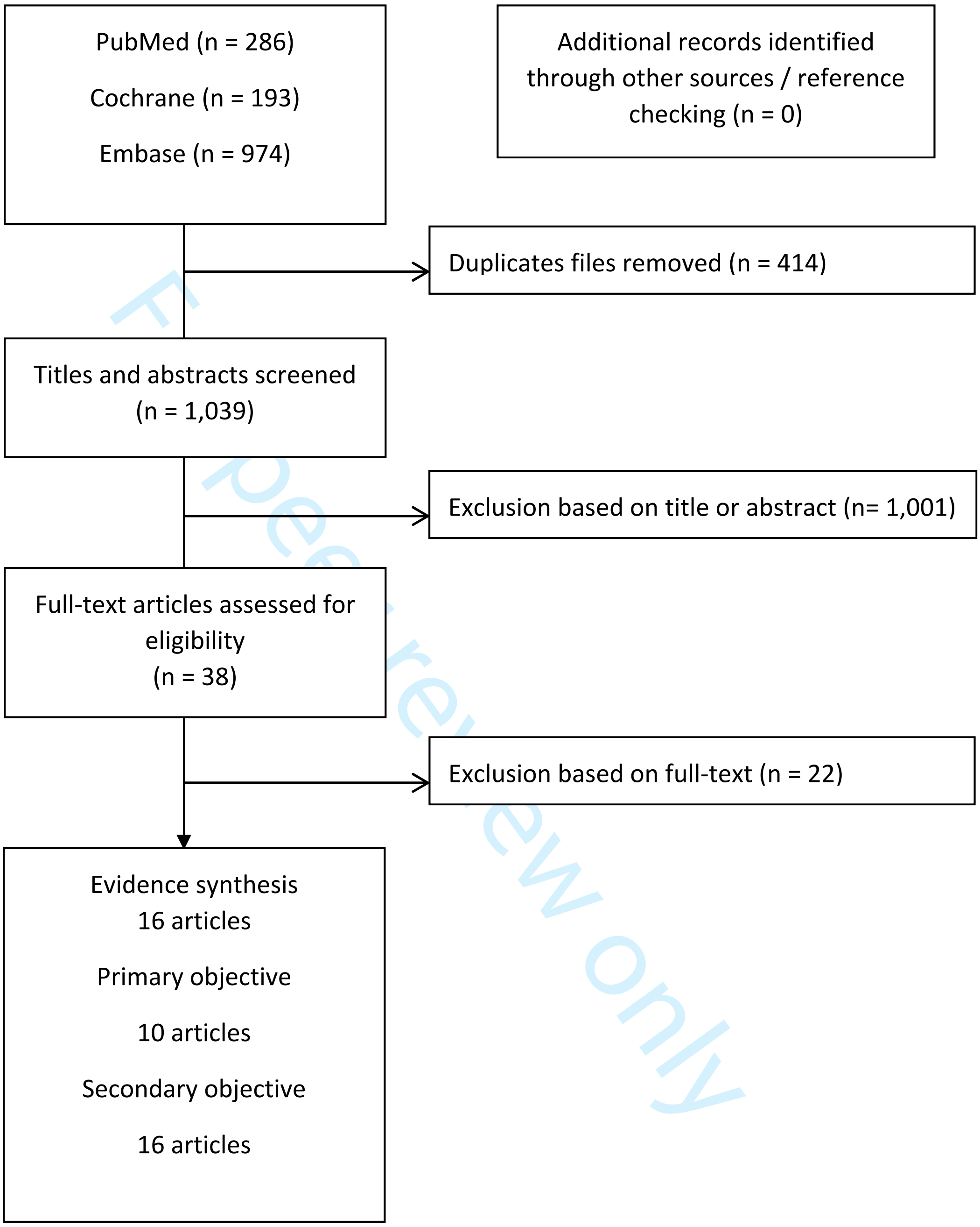

For peer review only

Supplementary File 3. Imputation of SD data from a previous meta-analysis

Tool	Ref.	Main author	Groups	N	SD	Pooled SD by study	Pooled SD overall
**IPSS**		**N = 12 studies**					
	49	Chapple	no data				
	73	Djavan	no data				
	147	Kirby	1	250	5.8	6.3	
			2	239	6.2		
			3	265	6.2		
			4	253	6.9		
	160	Lepor	no data				
	163	Lepor	no data				
	191	McConnell	no data				
	201	Mohanty	placebo	33	4	4.2	
			AB	36	4.4		
	208	Narayan	no data				
	254	Roehrborn	no data				
	255	Roehrborn	no data				
	262	Roehrborn	no data				
	305	van Kerrebroeck	N/A				
							**6.2**
**Q-max**		**N = 21 studies**					
	6	Abrams	N/A				
	33	Brawer	no data				
	50	chapple	no data				
	51	chapple	meta-analysis				
	53	christensen	N/A				
	79	elhilali	no data				
	101	gillenwater	no data				
	138	Kawabe	N/A				
	147	kirby	1	250	4.9		
			2	239	4.7	4.7	
			3	265	5.1		
			4	253	4.2		
	160	Lepor	no data				
	161	Lepor	no data				
	163	Lepor	no data				
	169	Lloyd	1	20	3.6		
			2	19	3.5	3.5	
			3	19	3.9		
			4	22	2.8		
	184	Martorana	N/A				
	201	Mohanty	placebo	33	2.6		
			AB	36	4.6	3.8	

For peer review only

	208	Narayan	no data				
	254	Roehrborn	no data				
	255	Roehrborn	no data				
	262	Roehrborn	no data				
	269	Schulman	N/A				
	305	van Kerrebroeck	N/A				
							**4.6**
**QoL**		**N = 5**					
	49	chapple	placebo	350	1		
			ocas 0.4	354	1.1	1.1	
			mr 0.4	699	1.1		
			ocas 0.8	706	1.1		
	160	Lepor	no data				
	254	Roehrborn	no data				
	255	Roehrborn	placebo	763	1.1		
			alfuzosin	759	1.1	1.1	
	305	van Kerrebroeck	N/A				
							**1.1**

In total, 12 studies assessed the IPSS, 21 assessed Q-max, and 5 assessed the IPSS QoL domain. Only studies that reported an SD at follow-up were included for the IPSS and Q-max data. No follow-up SDs were available for the QoL data, so baseline SDs were used. The SDs were imputed based on Table 6-35 from the NICE guideline on the management of LUTS [ref. 3].

Abbreviations: IPSS, International Prostate Symptom Score; N/A, article not accessible; Q-max, Peak urine flow rate; QoL, Quality of life; SD, standard deviation.

For peer review only

Supplementary File 4. Results of the risk of bias assessment

	Random sequence generation (selection bias)	Allocation concealment (selection bias)	Blinding of participants and personnel (performance bias)	Blinding of outcome assessment (detection bias)	Incomplete outcome data (attrition bias)	Selective reporting (reporting bias)	Other bias
Barkin et al	?	?	+	+	+	+	+
Debruyne et al	+	+	+	+	+	+	+
Fabricius	?	?	+	+	+	+	+
Gerber et al	−	−	−	−	−	+	+
Kaplan et al	−	−	−	−	+	+	+
Lee et al	+	+	−	−	−	+	+
Liaw & Kuo	−	?	−	−	?	+	?
Lin et al	+	?	−	−	+	+	+
Matsukawa	+	+	−	−	+	+	+
Yanardag et al	?	?	−	?	+	+	+

PRISMA 2009 Checklist

Section/topic	#	Checklist item	Reported on page #
**TITLE**			
Title	1	Identify the report as a systematic review, meta-analysis, or both.	1
**ABSTRACT**			
Structured summary	2	Provide a structured summary including, as applicable: background; objectives; data sources; study eligibility criteria, participants, and interventions; study appraisal and synthesis methods; results; limitations; conclusions and implications of key findings; systematic review registration number.	2
**INTRODUCTION**			
Rationale	3	Describe the rationale for the review in the context of what is already known.	4
Objectives	4	Provide an explicit statement of questions being addressed with reference to participants, interventions, comparisons, outcomes, and study design (PICOS).	4
**METHODS**			
Protocol and registration	5	Indicate if a review protocol exists, if and where it can be accessed (e.g., Web address), and, if available, provide registration information including registration number.	4
Eligibility criteria	6	Specify study characteristics (e.g., PICOS, length of follow-up) and report characteristics (e.g., years considered, language, publication status) used as criteria for eligibility, giving rationale.	5
Information sources	7	Describe all information sources (e.g., databases with dates of coverage, contact with study authors to identify additional studies) in the search and date last searched.	5
Search	8	Present full electronic search strategy for at least one database, including any limits used, such that it could be repeated.	Supplement
Study selection	9	State the process for selecting studies (i.e., screening, eligibility, included in systematic review, and, if applicable, included in the meta-analysis).	5-6
Data collection process	10	Describe method of data extraction from reports (e.g., piloted forms, independently, in duplicate) and any processes for obtaining and confirming data from investigators.	5-6
Data items	11	List and define all variables for which data were sought (e.g., PICOS, funding sources) and any assumptions and simplifications made.	5-6
Risk of bias in individual studies	12	Describe methods used for assessing risk of bias of individual studies (including specification of whether this was done at the study or outcome level), and how this information is to be used in any data synthesis.	6
Summary measures	13	State the principal summary measures (e.g., risk ratio, difference in means).	6
Synthesis of results	14	Describe the methods of handling data and combining results of studies, if done, including measures of consistency (e.g., I^2) for each meta-analysis.	6

For peer review only - http://bmjopen.bmj.com/site/about/guidelines.xhtml

PRISMA 2009 Checklist

Section/topic	#	Checklist item	Reported on page #
Risk of bias across studies	15	Specify any assessment of risk of bias that may affect the cumulative evidence (e.g., publication bias, selective reporting within studies).	6
Additional analyses	16	Describe methods of additional analyses (e.g., sensitivity or subgroup analyses, meta-regression), if done, indicating which were pre-specified.	NA
**RESULTS**			
Study selection	17	Give numbers of studies screened, assessed for eligibility, and included in the review, with reasons for exclusions at each stage, ideally with a flow diagram.	7
Study characteristics	18	For each study, present characteristics for which data were extracted (e.g., study size, PICOS, follow-up period) and provide the citations.	7 + supplement
Risk of bias within studies	19	Present data on risk of bias of each study and, if available, any outcome level assessment (see item 12).	7+ Supplement
Results of individual studies	20	For all outcomes considered (benefits or harms), present, for each study: (a) simple summary data for each intervention group (b) effect estimates and confidence intervals, ideally with a forest plot.	Figures
Synthesis of results	21	Present results of each meta-analysis done, including confidence intervals and measures of consistency.	8-12 + supplement
Risk of bias across studies	22	Present results of any assessment of risk of bias across studies (see Item 15).	7-9+supplement
Additional analysis	23	Give results of additional analyses, if done (e.g., sensitivity or subgroup analyses, meta-regression [see Item 16]).	NA
**DISCUSSION**			
Summary of evidence	24	Summarize the main findings including the strength of evidence for each main outcome; consider their relevance to key groups (e.g., healthcare providers, users, and policy makers).	12-13
Limitations	25	Discuss limitations at study and outcome level (e.g., risk of bias), and at review-level (e.g., incomplete retrieval of identified research, reporting bias).	13-14
Conclusions	26	Provide a general interpretation of the results in the context of other evidence, and implications for future research.	14
**FUNDING**			
Funding	27	Describe sources of funding for the systematic review and other support (e.g., supply of data); role of funders for the systematic review.	3

From: Moher D, Liberati A, Tetzlaff J, Altman DG, The PRISMA Group (2009). Preferred Reporting Items for Systematic Reviews and Meta-Analyses: The PRISMA Statement. PLoS Med 6(7): e1000097.

For peer review only - http://bmjopen.bmj.com/site/about/guidelines.xhtml

PRISMA 2009 Checklist

doi:10.1371/journal.pmed1000097 For more information, visit: www.prisma-statement.org. Page 2 of 2

For peer review only
